# Long-term gridded land evapotranspiration reconstruction using Deep Forest with high generalizability

**DOI:** 10.1038/s41597-023-02822-8

**Published:** 2023-12-18

**Authors:** Qiaomei Feng, Junyong Shen, Feng Yang, Shijing Liang, Jiang Liu, Xingxing Kuang, Dashan Wang, Zhenzhong Zeng

**Affiliations:** 1 School of Environmental Science and Engineering, Southern University of Science and Technology, Shenzhen, 518055 China; 2 Department of Computer Science and Engineering, Southern University of Science and Technology, Shenzhen, 518055 China; 3 Research Institute of Trustworthy Autonomous Systems, Southern University of Science and Technology, Shenzhen, 518055 China; 4 Guangdong Provincial Key Laboratory of Soil and Groundwater Pollution Control, Southern University of Science and Technology, Shenzhen, 518055 China

**Keywords:** Hydrology, Hydrology

## Abstract

Previous datasets have limitations in generalizing evapotranspiration (ET) across various land cover types due to the scarcity and spatial heterogeneity of observations, along with the incomplete understanding of underlying physical mechanisms as a deeper contributing factor. To fill in these gaps, here we developed a global Highly Generalized Land (HG-Land) ET dataset at 0.5° spatial resolution with monthly values covering the satellite era (1982–2018). Our approach leverages the power of a Deep Forest machine-learning algorithm, which ensures good generalizability and mitigates overfitting by minimizing hyper-parameterization. Model explanations are further provided to enhance model transparency and gain new insights into the ET process. Validation conducted at both the site and basin scales attests to the dataset’s satisfactory accuracy, with a pronounced emphasis on the Northern Hemisphere. Furthermore, we find that the primary driver of ET predictions varies across different climatic regions. Overall, the HG-Land ET, underpinned by the interpretability of the machine-learning model, emerges as a validated and generalized resource catering to scientific research and various applications.

## Background & Summary

Retrieving the long-term changes in evapotranspiration (ET) provides valuable insights into the biosphere-climate feedback and the intensity of land-atmosphere water circulations under global warming^1–3^. To date, a wide range of terrestrial ET products have been developed, featuring different spatial and temporal resolutions. These products include the Moderate Resolution Imaging Spectroradiometer (MODIS) Terrestrial ET (MOD16A2^4^), Breathing Earth System Simulator (BESS^5^), Penman-Monteith-Leuning model (PML_V2^6^), Global Land Evaporation Amsterdam Model (GLEAM^7^), Global Land Surface Satellite (GLASS^8^), Synthesized ET^9^, FLUXCOM^10^, FLUXNET-MTE^11^, ECMWF Reanalysis v5-Land (ERA5-Land^12^), and Global Land Data Assimilation System (GLDAS V2.1^13^), among others. Despite being widely used, these products tend to be sensitive to the choice of modeling approaches, encompassing both process-based models and empirical models. These methods may still grapple with limitations regarding their extrapolation abilities and parameter determination to varying degrees^14^, which persist in the challenge of precisely estimating terrestrial ET datasets. Importantly, there are few datasets that conduct self-assessment of their model’s generalizability and provide uncertainty quantifications alongside their ET estimations. This lack of assessment makes it unclear whether the inherent land surface heterogeneity is adequately captured by the model.

Furthermore, empirical relationships derived from statistical models based on eddy-covariance flux observations generally outperform process-based models, mainly due to the latter’s underutilization of forcing information and intricate model structures^15^. Nevertheless, there have been limited efforts to construct global datasets using empirical models guided by flux observations, with FLUXCOM being a notable exception. The FLUXCOM dataset has utilized data from the FLUXNET La Thuile synthesis dataset and the CarboAfrica network sites^16^, and leverages a diverse array of machine-learning (ML) algorithms, including Random Forest, Artificial Neural Network, Multivariate Adaptive Regression Splines, Model-Tree Ensemble, Kernel Ridge Regression, and Support Vector Regression^10^. FLUXCOM employs various ML methods to potentially capture nonlinear relationships within observations, showing promise in improving the performance and accuracy in ET reconstruction^16,17^. Yet, the Deep Forest algorithm, a recently emerged and powerful tool, has remained unexplored in this context. The Deep Forest, which is a tree-based ensemble model, combines the strengths of Random Forest and deep learning^18^. Inheriting the layer-by-layer nature of deep neural networks, the Deep Forest simplifies the often tedious hyperparameter tuning process^17^ and automatically determines the optimal model complexity based on the characteristics of input data, ensuring robust generalizability^18^. The Deep Forest approach shows promising potential for capturing the nonlinear relationships among inputs, offering an alternative to deep neural networks for the reason that Deep Forest efficiently avoids overfitting even with limited training data^18–25^. Thus, with Deep Forest as a state-of-the-art method and the abundance of observational, meteorological, and remotely sensed data, there exists an opportunity to develop highly accurate and long-term ET datasets since the satellite era.

In this study, we utilize Deep Forest to generate the highly generalized land (HG-Land) ET dataset at a 0.5° resolution, spanning from 1982 to 2018. Our approach integrates meteorological data from ECMWF Reanalysis v5-Land (ERA5-Land^26^) and Climatic Research Unit gridded Time Series dataset (CRU TS v4.05^27^), satellite data from NOAA Climate Data Record (CDR) of AVHRR Leaf Area Index (LAI) and Fraction of Absorbed Photosynthetically Active Radiation (FAPAR) v5.0^28^, and *in-situ* observational data from FLUXNET2015^29^. We validated the performance of HG-Land ET using both *in-situ* observations and the Conserving Land–Atmosphere Synthesis Suite (CLASS^30^) dataset, allowing for an evaluation of HG-Land at both the site and basin scale. The CLASS is a robust comprehensive dataset that concurrently balances both water and energy budgets while also quantifying uncertainties in each water component^30^. This dataset provides continuous ET estimations at a 0.5° resolution, spanning from 2003 to 2009. The ET component within the CLASS was derived from the DOLCE ET dataset, amalgamating versions 2A, 2B, and 3A of the GLEAM, FLUXNET-MTE, MOD16, and PML datasets^31^.

Furthermore, we employed the SHapley Additive exPlanations (SHAP^32^) and Accumulated Local Effects (ALE^33^) plots to gain insights into the main factors driving ET predictability and to find how ET estimations respond to changes in the predictors. The SHAP method, rooted in cooperative game theory, employs Shapley values^34^ to fairly attribute the model’s predictions to the coalitional features while considering their interactions. The ALE plot, developed by Daniel W. Apley and Jingyu Zhu, is tailored to visualize how predictors impact predictions in supervised learning while maintaining computational efficiency^33^. Moreover, ALE exhibits robustness even when features are strongly correlated, a common scenario encountered in practical applications^33^. Our findings demonstrate that the HG-Land ET product adeptly captures the magnitude of ET, as well as its spatiotemporal patterns and seasonal variability. Moreover, the dataset exhibits satisfactory generalizability, as validation results confirm. Overall, constructing the ET datasets enriches our understanding of the interactions between the atmosphere and surface land type characteristics.

## Methods

### Datasets

#### Forcing datasets

Here we incorporated biophysical and meteorological information into the forcing datasets to develop a long-term terrestrial ET product. All gridded datasets were aggregated into monthly values with a consistent 0.5° resolution. The vegetation biophysical information including LAI and FAPAR was collected from a daily 0.05° resolution product NOAA CDR AVHRR LAI and FAPAR v5.0^28^, which is obtained from NOAA National Centers for Environmental Information. The missing values were filled by the multi-year monthly means of LAI and FAPAR.

The meteorological inputs were collected from ERA5-Land^26^ and CRU TS v4.05^27^. We used the monthly averaged fields of surface net solar radiation, 10 m wind speed, skin temperature, and surface pressure from the ERA5-Land dataset which originally has 0.1° resolution, and can be accessed from the Copernicus Climate Change Service (C3S) Climate Data Store. We obtained 2 m temperature, precipitation, vapor pressure, potential ET, wet days, and frost days from the CRU TS v4.05 dataset. To gain a deeper understanding of the potential uncertainties associated with the selection of forcing datasets, we altered the sources of input variables and subsequently compared the resulting discrepancies (see Supplementary text; Supplementary Tables 1, 2; Supplementary Figs. 1, 2).

We utilized *in-situ* ET observations collected from FLUXNET2015^29^ dataset as model input. The FLUXNET2015 dataset provides surface fluxes at a fine temporal resolution of half-hourly and hourly, spanning the period from 1992 to 2014. For this study, we selected monthly observations from 195 flux tower sites representing 11 IGBP (International Geosphere-Biosphere Programme) surface land classifications. To address missing data in the observations, we employed the widely accepted Marginal Distribution Sampling (MDS) method^35^. Additionally, we applied the energy correction to ensure the energy balance closure^15^.

#### Validation datasets

The HG-Land ET dataset was validated using both site-scale observations and basin-scale measurements. At the site scale, we evaluate the model results using ET observations obtained from the FLUXNET2015 dataset. For basin-scale validation, despite the widespread recognition of the water budget equation (Eq. 1), it tends to face challenges related to incomplete budget closure when combining multi-source datasets for each budget term^30,36–38^.

1 $${ET}_{WB}=P-Q-TWSC$$

where *P* and *Q* are the precipitation and runoff for a given basin, and *TWSC* refers to the terrestrial water storage changes, respectively. To ensure the integrity of the water budget closure, as shown in Eq. 2, we utilized the ET component within the CLASS dataset as a benchmark for assessing the basin-scale performance of HG-Land.

2 $${ET}_{WB}\pm {un}_{ET}=(P\pm {un}_{P})-(Q\pm {un}_{Q})-(TWSC\pm {un}_{TWSC})$$

where *un* represents the uncertainties associated with each component.

### ET reconstruction method

**Fig. 1:**
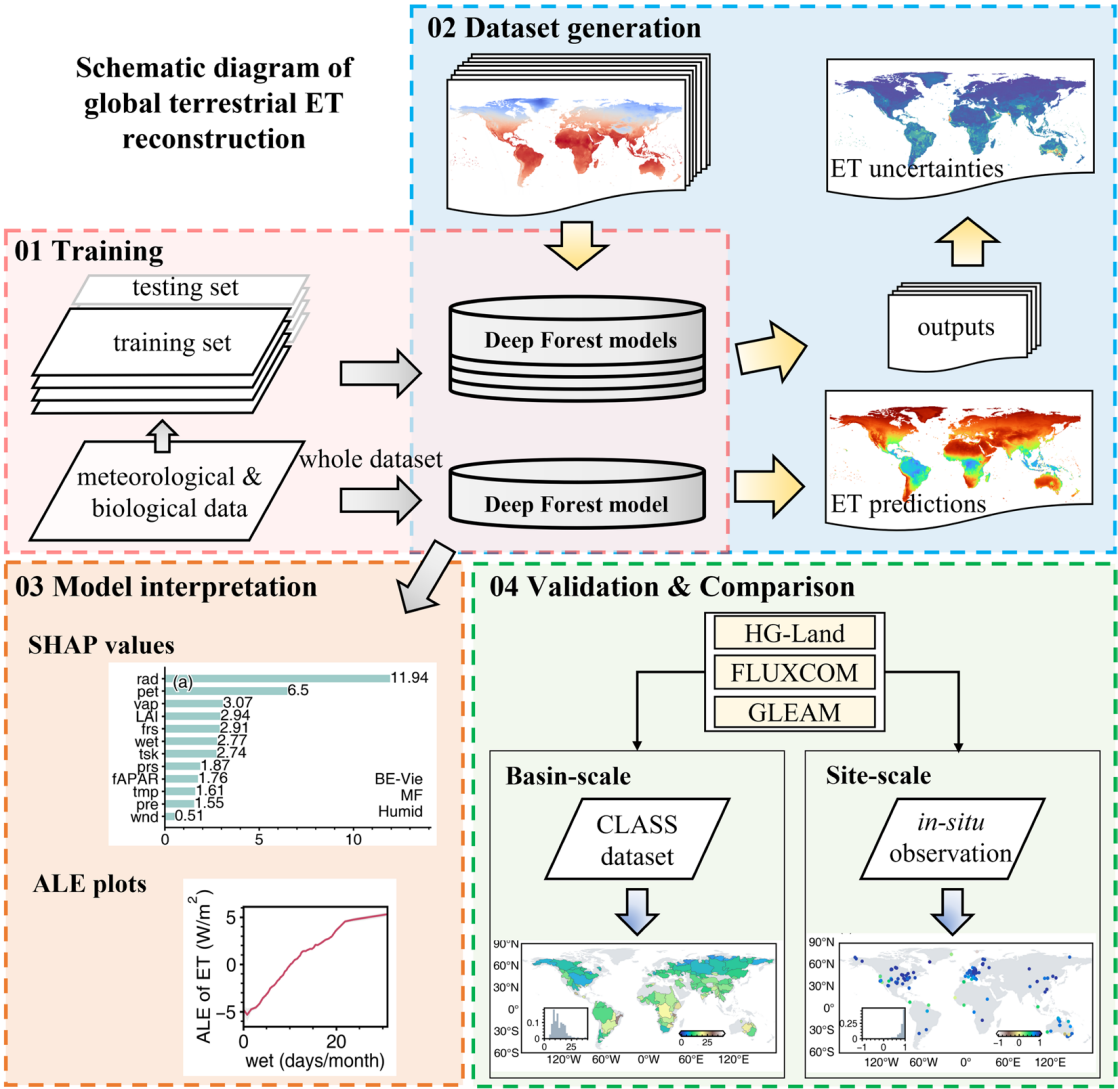
Workflow for reconstructing the ET dataset.

The procedure of developing the HG-Land ET dataset consists of multiple stages, including model development, data reconstruction, model interpretation, validation, and comparison with current products. A comprehensive workflow of our method is depicted in Fig. 1.

#### Model development

In our study, we set up each cascade layer (Fig. S3) within the model to include 8 Random Forest regressions and 8 Extra Trees regressions, with each forest comprising a total of 100 trees. The prerequisite minimum sample count for note splitting is set at 2. The number of cascade layers is automatically determined based on whether the validation performance at the new cascade layer surpasses the performance at the previous layers, providing flexibility to adjust the model structure in response to the input data.

We aligned forcing datasets with *in-situ* ET observations, resulting in a dataset of 10,976 samples consisting of 12 meteorological and vegetation variables (Table 1). The dataset generation process comprises two key steps: the assessment of uncertainties in predicted ET resulting from model extrapolation, and the generation of the final ET estimations. The samples were first partitioned into a training set, encompassing 10 different surface land classifications, and the remaining distinct surface land classification was reserved for the testing set to be used for validation. This partitioning approach was consistently applied across each distinct surface land classification, yielding a total of 11 different combinations of training and testing sets, as shown in Table 2. In the initial step, model training was conducted using different training sets, thereby generating 11 global estimated ET outputs. The performances of the model on each testing set and at each site were consolidated in Figs. S4, S5. We then assessed the estimated uncertainties associated with the model extrapolation by computing the standard deviation across the 11 global ET outputs (Fig. S6). Subsequently, the final dataset was generated by using the entire set of data samples. These two steps result in the HG-Land dataset, which includes global ET estimations and quantifies the associated monthly uncertainties stemming from model extrapolation. The generation process was facilitated through the utilization of the Deep Forest (DF21) python package^18^.

**Table 1:** Input data sources for Deep Forest model development.

Datasets	Variable	Function
CRU TS v4.05	2 m temperature (tmp, degree Celsius), precipitation (pre, mm/month), vapour pressure (vap, hPa), potential ET (pet, mm/day), wet days (wet, days/month), frost days (frs, days/month)	meteorological information
ERA5-Land	surface net solar radiation (rad, J/m^2^), 10 m wind speed (wnd, m/s), skin temperature (tsk, K), surface pressure (prs, Pa)	meteorological information
NOAA CDR AVHRR LAI and FAPAR v5.0	fAPAR (–), LAI (m^2^/ m^2^)	biological information

**Table 2:** Composition of testing set samples in different land cover types.

Testing sets	Land cover type	Number of samples in the testing set
1	CRO	1295
2	CSH	135
3	DBF	1562
4	EBF	821
5	ENF	3161
6	GRA	1801
7	MF	529
8	OSH	417
9	SAV	282
10	WET	528
11	WSA	445

Note: the land cover classifications defined by the IGBP (International Geosphere Biosphere Programme) include CRO (croplands), CSH (closed shrublands), DBF (deciduous broadleaf forests), EBF (evergreen broadleaf forests), ENF (evergreen needleleaf forests), GRA (grasslands), MF (mixed forests), OSH (open shrublands), SAV (savannas), WET (permanent wetlands), and WSA (woody savannas).

#### Model interpretation

In this study, we exploited the SHAP method^32^ and ALE plots^33^ to provide an interpretation of the model, leveraging their solid theoretical foundations and wide applicability across various fields^39–44^. SHAP values are calculated for each sample in the dataset to quantitatively capture the impacts of each feature within that sample. Higher positive SHAP values indicate a greater positive impact on the prediction and vice versa^32^. Therefore, the importance of a feature is determined by the magnitude of its SHAP value, specifically the absolute value. Given that feature contribution may vary across samples in the dataset, the mean absolute SHAP values were aggregated across a subset of samples to represent the importance of each feature within that subset. To gain insights into whether distinct main factors influencing ET estimations vary across different climates, we chose 20 sites characterized by either humid or dry climatic conditions, as defined by aridity index (AI) thresholds provided by UNEP^45^ (Table S3). The AI data for each site was obtained from the Global-AI_PET_v3 dataset^46^ (Table S4). We then computed SHAP values for each site individually across its respective set of samples.

To investigate the impact of features on model predictions, we also employed the ALE method. This technique assesses the influence of features across distinct intervals, aggregating their local effects within each interval to yield valuable insights into the alteration of the model’s predictions as the feature values fluctuate within specific ranges^33^. The ALE was computed using the entire dataset’s samples, with each feature value range partitioned into 50 intervals.

### Dataset evaluation

#### Assessment at both site and basin scales

The performance of the HG-Land ET dataset was evaluated through two distinct approaches: site-scale comparisons against *in-situ* observations and basin-scale comparisons using ET data from the CLASS dataset as references. For the site-scale assessments, we validated the accuracy of HG-Land ET across 195 distinct flux tower sites sourced from the FLUXNET2015 dataset. Furthermore, we extended the validation on the 11 land cover classifications, categorized based on the corresponding surface land types of all available sites. For the basin-scale evaluation, our analysis encompassed 121 global major river basins, each exceeding 100,000 km^2^. Within each basin, we employed statistical metrics to assess accuracy.

#### Evaluation metrics

We conducted data evaluation at both the local site and broader basin scales. The evaluation process employed four key metrics for comprehensive analysis, including the Pearson correlation coefficient (R), Root Mean Square Error (RMSE), Mean Absolute Error (MAE), and Relative Bias (RB). These metrics are defined as follows, respectively:

3 $$R=\frac{{\sum }_{i=1}^{n}({y}_{pre,i}-\bar{{y}_{pre}})({y}_{true,i}-\bar{{y}_{true}})}{\sqrt{{\sum }_{i=1}^{n}{({y}_{pre,i}-\bar{{y}_{pre}})}^{2}}\sqrt{{\sum }_{i=1}^{n}{({y}_{true,i}-\bar{{y}_{true}})}^{2}}}$$

4 $$RMSE=\sqrt{\frac{1}{n}{\sum }_{i=1}^{n}{({y}_{pre,i}-{y}_{true,i})}^{2}}$$

5 $$MAE=\frac{1}{n}{\sum }_{i=1}^{n}|{y}_{pre,i}-{y}_{true,i}|$$

6 $$RB=\frac{{\sum }_{i=1}^{n}({y}_{pre,i}-{y}_{true,i})}{{\sum }_{i=1}^{n}{y}_{true,i}}\times 100 \% $$

where *i* is the *i*^*th*^ sample; *n* is the total number of samples; *y*_*true,i*_ is the *i*^*th*^ measured ET; and *y*_*pre,i*_ represents the *i*^*th*^ estimated ET. Superior dataset performance is indicated by a higher R value, lower RMSE, lower MAE, and reduced absolute RB value.

### Comparison with other products

To thoroughly assess the accuracy of the HG-Land ET dataset, we conducted comparisons with two widely used global datasets. The first dataset, FLUXCOM^10^, is constructed from a combination of remote sensing data and meteorological data sourced from CRUNCEP_v8^47^, utilizing different ML methods and incorporating three energy balance closure corrections. The FLUXCOM dataset spans from 1982 to 2016 with monthly frequency and a 0.5° resolution. The second is the GLEAM v3.6a^7^ dataset, which offers monthly data with a resolution of 0.25° and covers the period from 1980 to 2021. The GLEAM dataset was aggregated to 0.5° resolution for further analyses. Specifically, we examined the long-term averaged spatial patterns and assessed global annual trends of all ET products during the shared period of 1982–2016, and delved into the seasonal fluctuations across four major Köppen climate classifications^48^. Moreover, to ensure comprehensive comparisons among these datasets at the site scale, we meticulously selected 11 representative flux tower sites, each corresponding to distinct land types. This selection process considered the duration of their respective data records. Additionally, at the basin scale, we identified 6 representative basins from different continents and conducted a time series analysis for the period of 2003–2009, aligning with the available record period of the reference dataset CLASS.

## Data Records

The HG-Land ET dataset^49^ is now available for access through the Science Data Bank (10.57760/sciencedb.10519). This product provides global monthly data on terrestrial ET, measured in W/m^2^, with a spatial resolution of 0.5°. It covers the period from January 1982 to December 2018. The data is stored in a network Common Data Form (netCDF) format, conveniently contained within a single file. This file comprises five variables namely: time, longitude, latitude, ET, and ET standard deviation.

## Technical Validation

### Statistical accuracy assessment

**Fig. 2:**
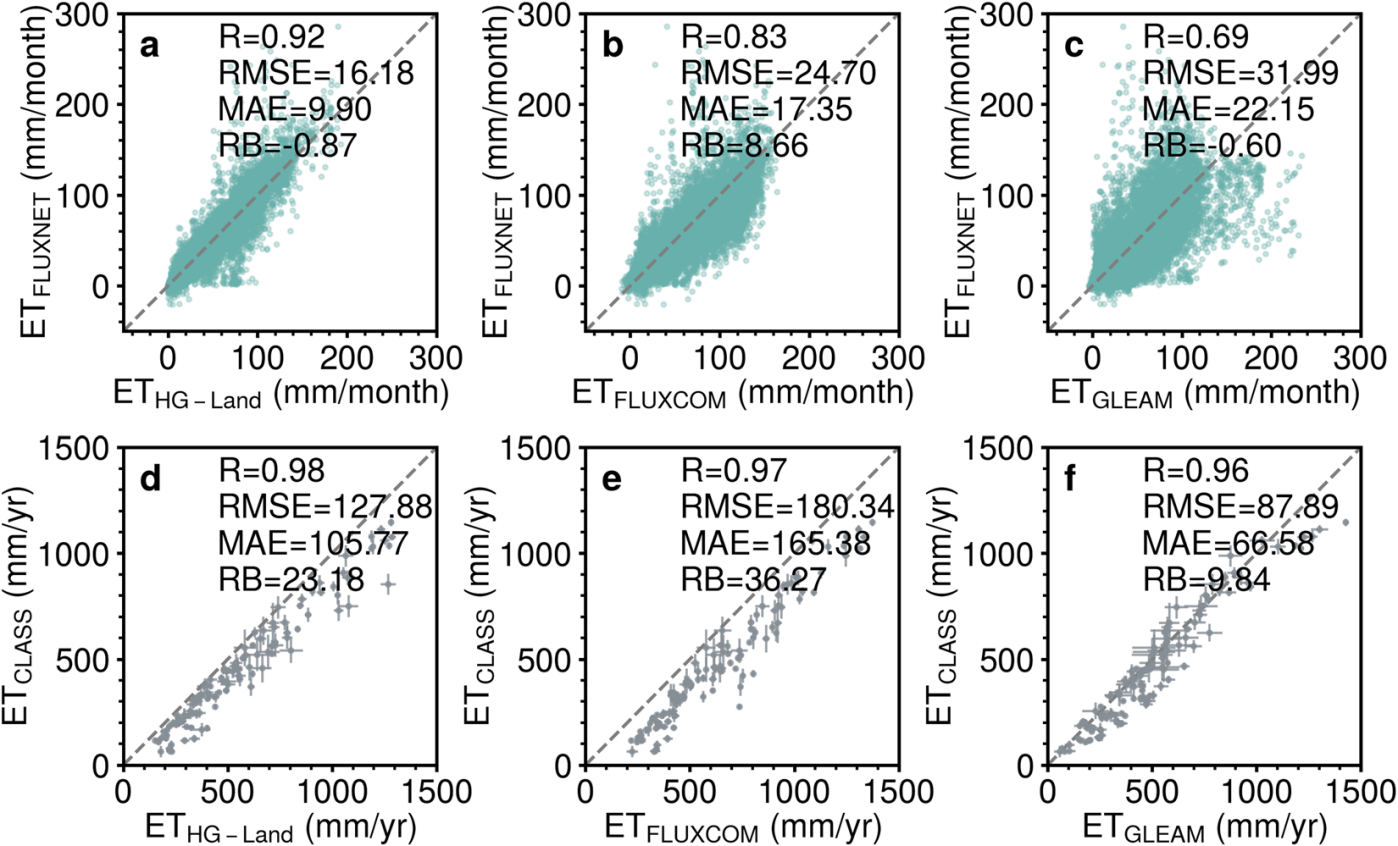
Comparison of monthly ET from FLUXNET2015 (1992–2014) and mean annual ET from CLASS (2003–2009) with ET estimations from three datasets. (**a,d**) HG-Land; (**b,e**) FLUXCOM; and (**c,f**) GLEAM. To ensure a fair comparison, the site-scale comparison was conducted using a sample size of 10,475, after excluding missing values in the GLEAM dataset.

**Fig. 3:**
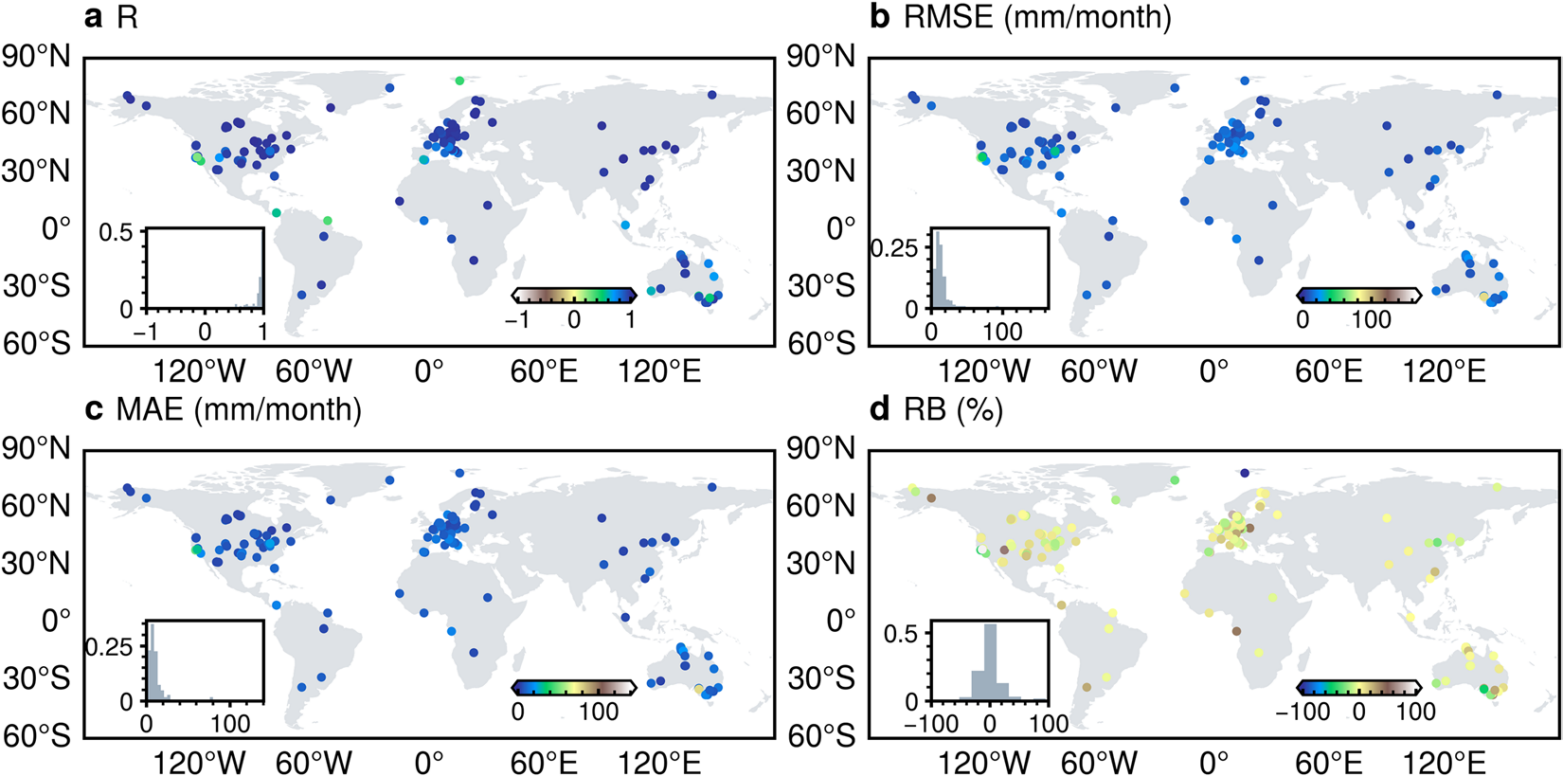
Spatial variability of HG-Land ET accuracy across different sites compared to FLUXNET observations. (**a**) R; (**b**) RMSE; (**c**) MAE; (**d**) RB. The inset figure shows the frequency distribution histogram of each metric. The flux sites US-Wi1, US-Wi7, and US-Wi9 were omitted from the figure due to a limited availability of valid data records from these sites.

Compared to the site-scale observations, the HG-Land ET dataset shows an overall accuracy characterized by R, RMSE, MAE, and RB values of 0.92, 16.18 mm/month, 9.90 mm/month, and −0.87%, respectively (Fig. 2a). We further investigated the accuracy of the HG-Land ET dataset at individual sites (Fig. 3), excluding samples from sites with fewer than two valid data records from the metric calculations. Across most sites, RMSE and MAE values primarily remain below 20 mm/month, although there are instances of lower accuracy at a few sites, particularly located in Southeastern Australia, Western US, and the Arctic Ocean.

**Fig. 4:**
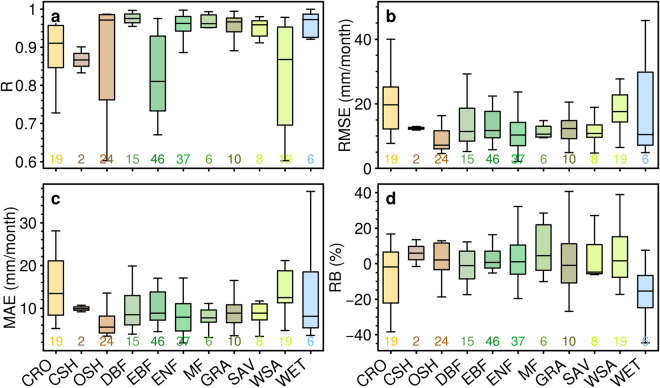
Box plots for the accuracy of HG-Land ET across 11 land cover classifications. (**a**) R; (**b**) RMSE; (**c**) MAE; (**d**) RB. The colored numbers indicate the number of sites included in each corresponding box. Outliers are not shown in the plots.

The site-scale estimations were further categorized and evaluated based on IGBP land classifications, as depicted in Fig. 4. The box plots corresponding to sites categorized as croplands (CRO), woody savannas (WSA), and permanent wetlands (WET) land types display elongated boxes, indicating a broader range of accuracy variability in ET estimations for these specific site categories, as measured by RMSE and MAE metrics (Fig. 4b,c). In contrast, other classifications have relatively modest RMSE and MAE values with narrower data dispersion. Additionally, the closed shrublands (CSH), open shrublands (OSH), and mixed forests (MF) land types predominantly exhibit positive RB values, while the WET land type predominantly demonstrates negative values (Fig. 4d). This suggests a tendency for the dataset to potentially overestimate or underestimate ET values for sites belonging to these land types, respectively.

**Fig. 5:**
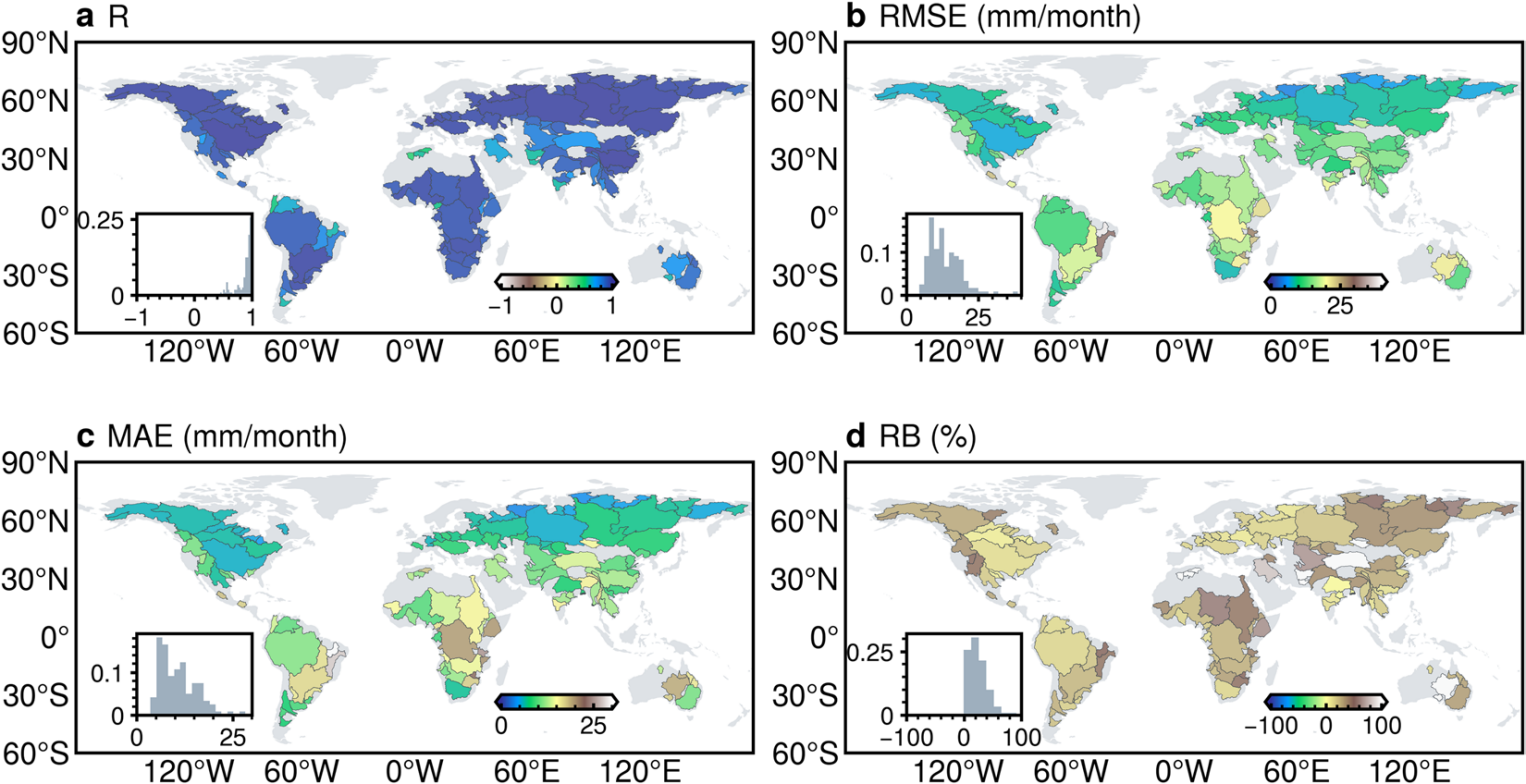
Spatial variability of HG-Land ET accuracy over basins compared to CLASS ET. (**a**) R; (**b**) RMSE; (**c**) MAE; (**d**) RB. The inset figure shows the frequency distribution histogram of each metric.

We also conducted a comparison between the monthly estimations of HG-Land ET and the ET data from the CLASS dataset at the basin scale. The overall accuracy of the HG-Land ET dataset is quantified by a high R-value of 0.98 (Fig. 2d). Notably, the HG-Land dataset has good accuracy in the Northern Hemisphere (NH), evident from the relatively low RMSE and MAE values (Fig. 5b,c) within this region. However, in the Southern Hemisphere (SH), the accuracy of HG-Land is slightly lower, primarily due to the limited availability of flux sites in the SH. Additionally, across various basins, all RB values exhibit positively, implying that the estimations provided by HG-Land are generally higher than the corresponding values of the CLASS dataset.

### Model interpretability

The SHAP and ALE methods are integrated to conduct a comprehensive investigation into the impact contributions of 12 variables (Table 1) on ET model prediction. The SHAP values displayed in Figs. S7, 8 facilitated a comparison of feature contributions between sites in dry and humid regions. Additionally, the ALE plots in Fig. S9 were employed to visually depict the effects of each variable on ET, providing insights into their behavior over specific intervals.

Across all the selected sites, as shown in Figs. S7, 8, the key influencers on ET estimations and the patterns of feature contributions exhibit variability from site to site. For example, at sites BE-Vie (Fig. S7a) and AU-Cum (Fig. S8a), the dominant driver for ET predictions is the rad variable, with the contributions of all other features notably lower in comparison. In contrast, at some sites, the contributions of features are more balanced, exemplified by CG-Tch (Fig. S7e), PA-SPs (Fig. S7j), AU-DaS (Fig. S8b), and AU-Stp (Fig. S8d). Furthermore, among sites located in humid regions, 7 out of 10 have rad as the main driver. This finding aligns with previous research that identified the importance of surface net solar radiation in determining ET^50–52^. In sites within dry climates, both rad and LAI emerge as the two principal drivers. Additionally, LAI takes the role of the main driver for all sites classified under the arid climate subtype (Fig. 8e,g,i), consistent with prior studies indicating that higher LAI enhances ET, especially in dry regions^53^. Furthermore, rad remains a significant contributor to ET in dry climates, particularly in semi-arid and dry subhumid regions.

The ALE plots of ET are visually represented in Fig. S9. The effects of variables on ET can be categorized into two groups: those that consistently influence ET through the entire range of values (Figs. S9c–h,k,l) and those whose impact is restricted to certain parts of their numerical intervals, with negligible effects on ET outside of those ranges (Fig. S9a,b,i,j). Notably, we observed that as LAI increases, ET estimations exhibit a steeper rise until LAI reaches around 1.5 m^2^/m^2^, after which the rate of ET increase decelerates. This observation potentially explains why the effect of LAI predominates in arid regions, as indicated by the results of SHAP values (Fig. S8e,g,i).

Consequently, the integration of SHAP and ALE methods furnishes valuable insights into the model’s behavior in generating results. Furthermore, it presents an opportunity to uncover previously unknown relationships within the data, thereby enriching our understanding of the land-atmosphere interactions.

### Comparison with existing products

#### Statistical accuracy comparison

We conducted a comprehensive comparison between the HG-Land ET dataset and two widely used ET datasets, namely FLUXCOM^10^ and GLEAM v3.6a^7^. Our validation was performed at both the site and basin scales. Our analysis demonstrated that the HG-Land ET outperforms FLUXCOM and GLEAM in terms of accuracy, both in the context of overall accuracy as measured by statistical metrics (Fig. 2a–c) and accuracy at individual sites (Fig. 3 versus Figs. S10, S11). Specifically, the FLUXCOM and GLEAM datasets display an overall performance of 0.83 and 0.69 for R, respectively, which are notably lower in comparison to the 0.92 achieved by HG-Land ET (Fig. 2a–c). Moreover, when considering the values of RMSE, MAE, and RB, both FLUXCOM and GLEAM present higher absolute values than those observed for HG-Land ET dataset.

**Fig. 6:**
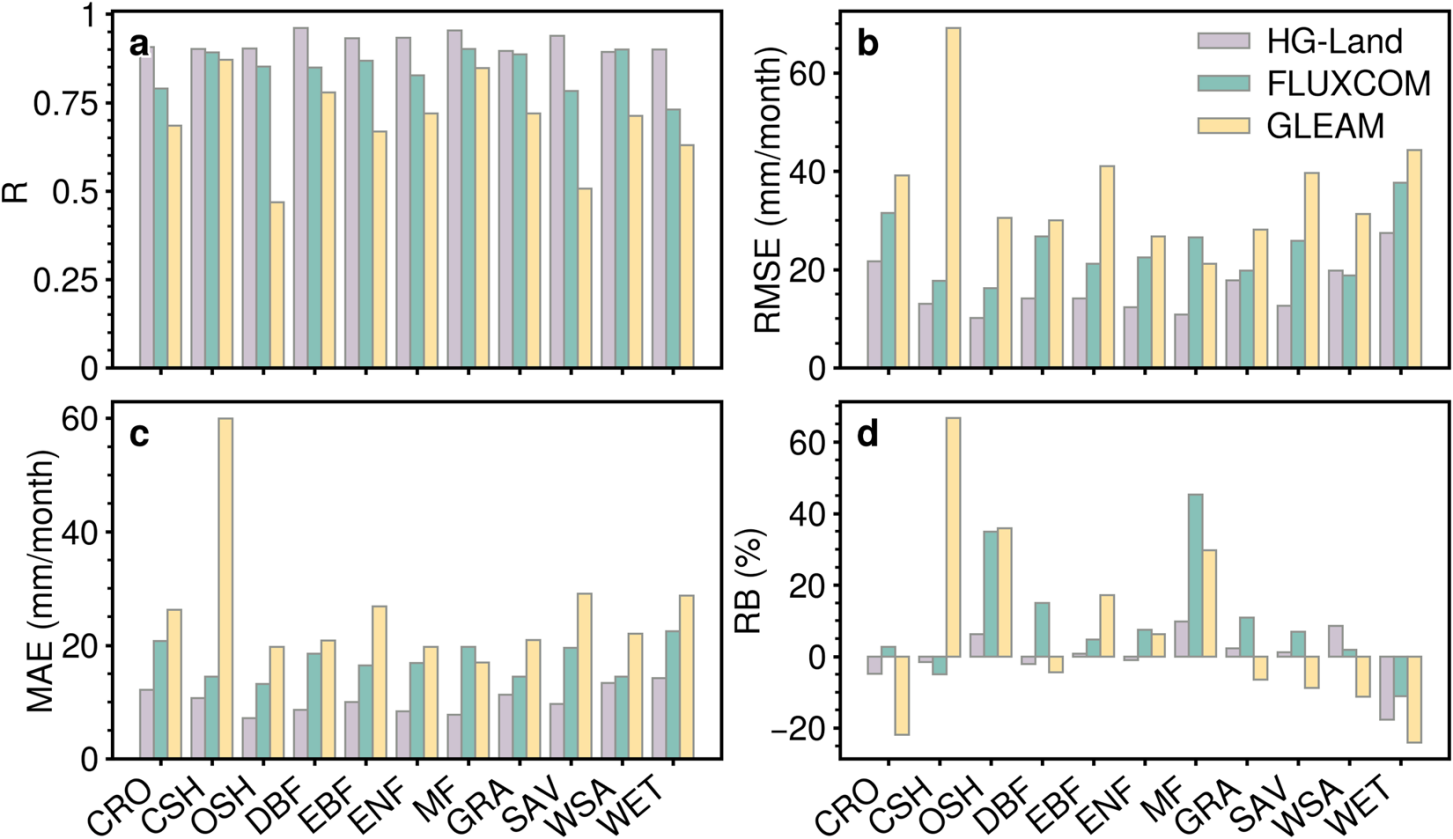
Comparison of accuracy among HG-Land, FLUXCOM, and GLEAM across 11 land cover classifications. (**a**) R; (**b**) RMSE; (**c**) MAE; (**d**) RB.

Furthermore, the comparison of the three datasets at the site scale was conducted based on IGBP land classifications. Overall, HG-Land consistently demonstrates higher R values and diminishes bias across almost all land cover classifications (Fig. 6). Notably, sites located in regions with WET land cover exhibit relatively high RMSE and MAE values, along with negative RB values across all three datasets (Fig. 6b-d). This suggests that the ET values recorded by flux sites falling within the WET land classification are inadequately captured and underestimated by all three datasets. Additionally, these datasets consistently tend to overestimate ET values for sites situated in areas characterized by MF and OSH land cover.

Given that the ET component in the CLASS dataset stems from the amalgamation of three GLEAM datasets (v2a, v2b, and v3) through the DOLCE product^31^, a cautious approach is necessary when interpreting the basin-scale validations, considering the use of the GLEAM dataset for comparison. The mean annual estimations of the three products compared to CLASS are summarized in Fig. 2d-f. Both HG-Land and FLUXCOM consistently tend to overestimate basin-scale ET, with RB values of 23.18% and 36.27%, respectively (Fig. 2d,e). However, HG-Land displays a relatively lower bias compared to FLUXCOM in terms of RMSE and MAE. Conversely, GLEAM demonstrates better performance at the basin scale compared to the other two datasets, evident from its closer alignment with the 1:1 line. In terms of spatial patterns, HG-Land showcases its highest performance in the mid-high latitudes and Amazon basins when compared to FLUXCOM and GLEAM (Fig. 5 versus Figs. S12,S13). FLUXCOM exhibits relatively improved performance in the mid-high latitudes of the NH compared to its performance in the tropical and subtropical areas (Fig. S12b,c). On the other hand, GLEAM performs well in tropical and subtropical regions (Fig. S13b,c), though it tends to overestimate in the high latitudes of the NH (Fig. S13d).

#### Spatial and temporal variations

**Fig. 7:**
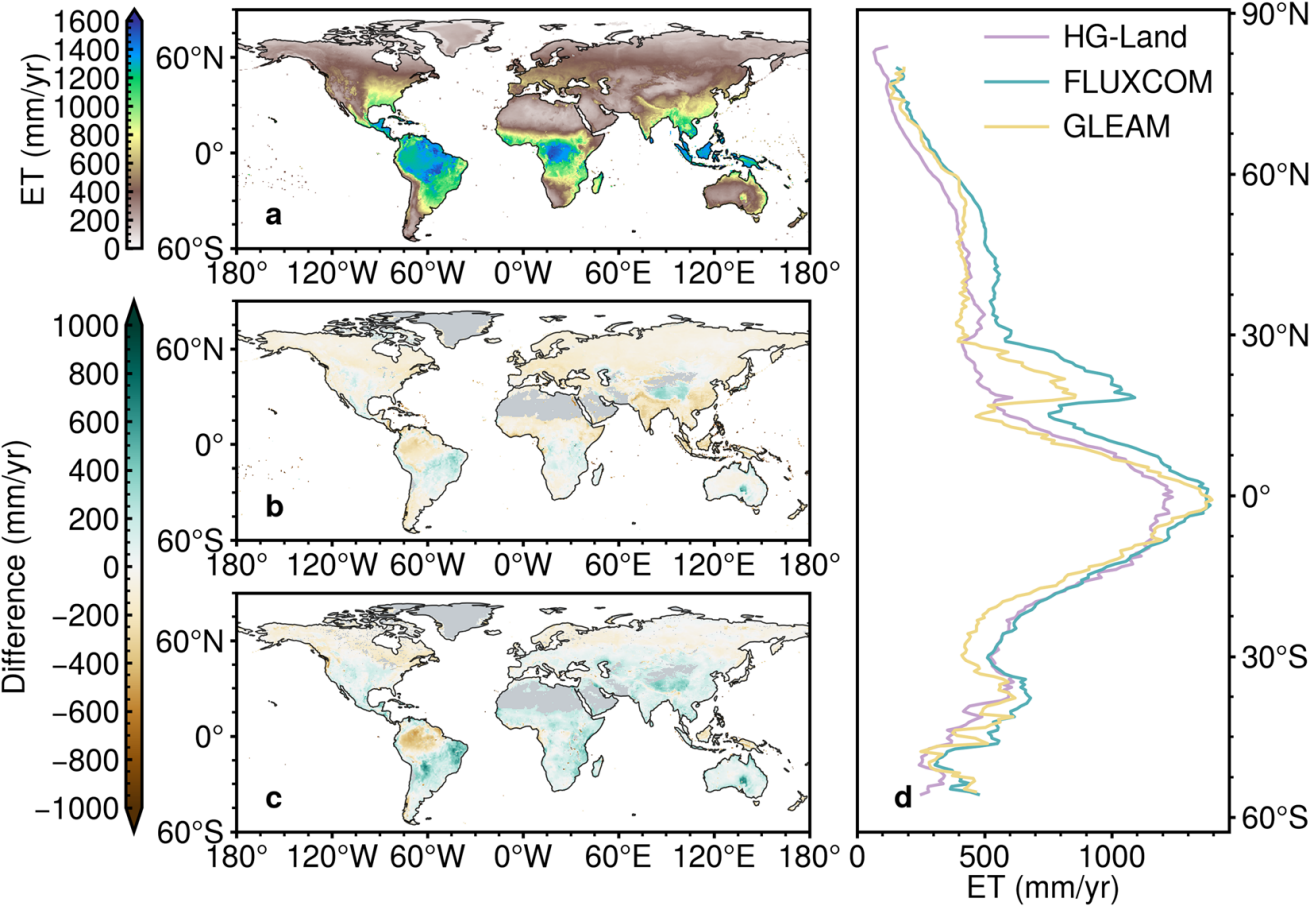
Comparison of global and zonal distribution of multi-year average ET. Spatial patterns of multi-year average ET for (**a**) HG-Land and the difference between HG-Land and (**b**) FLUXCOM, as well as the difference between HG-Land and (**c**) GLEAM from 1982 to 2016. Non-vegetated areas are masked in gray, based on the FLUXCOM pattern. (**d**) Comparison of zonal-averaged ET among HG-Land, FLUXCOM, and GLEAM.

The spatial pattern of the HG-Land ET dataset was further meticulously contrasted with that of the FLUXCOM and GLEAM ET datasets, focusing on their mean annual values. Our analysis revealed that, during the period from 1982 to 2016, tropical regions exhibit the highest ET values, while high latitudes show the lowest values across all three products (Fig. 7d). FLUXCOM particularly stands out by displaying the highest values along the zonal direction, a piece of evidence further corroborated across four major Köppen climate regions (Fig. S14). However, discernible disparities emerge between the HG-Land ET dataset and the other two ET datasets. Specifically, HG-Land ET displays higher values primarily in Southeast Brazil, Southern Australia, and the Tibetan Plateau, while lower values are observed in Amazon Basin, Southern Asia, Southeast Asia, and Europe in comparison to FLUXCOM. Meanwhile, HG-Land ET values are lower than GLEAM mainly in tropical regions like the Amazon Basin and Southeast Asia, but higher values occurred in the remainder of the region from 30°S to 45°N.

**Fig. 8:**
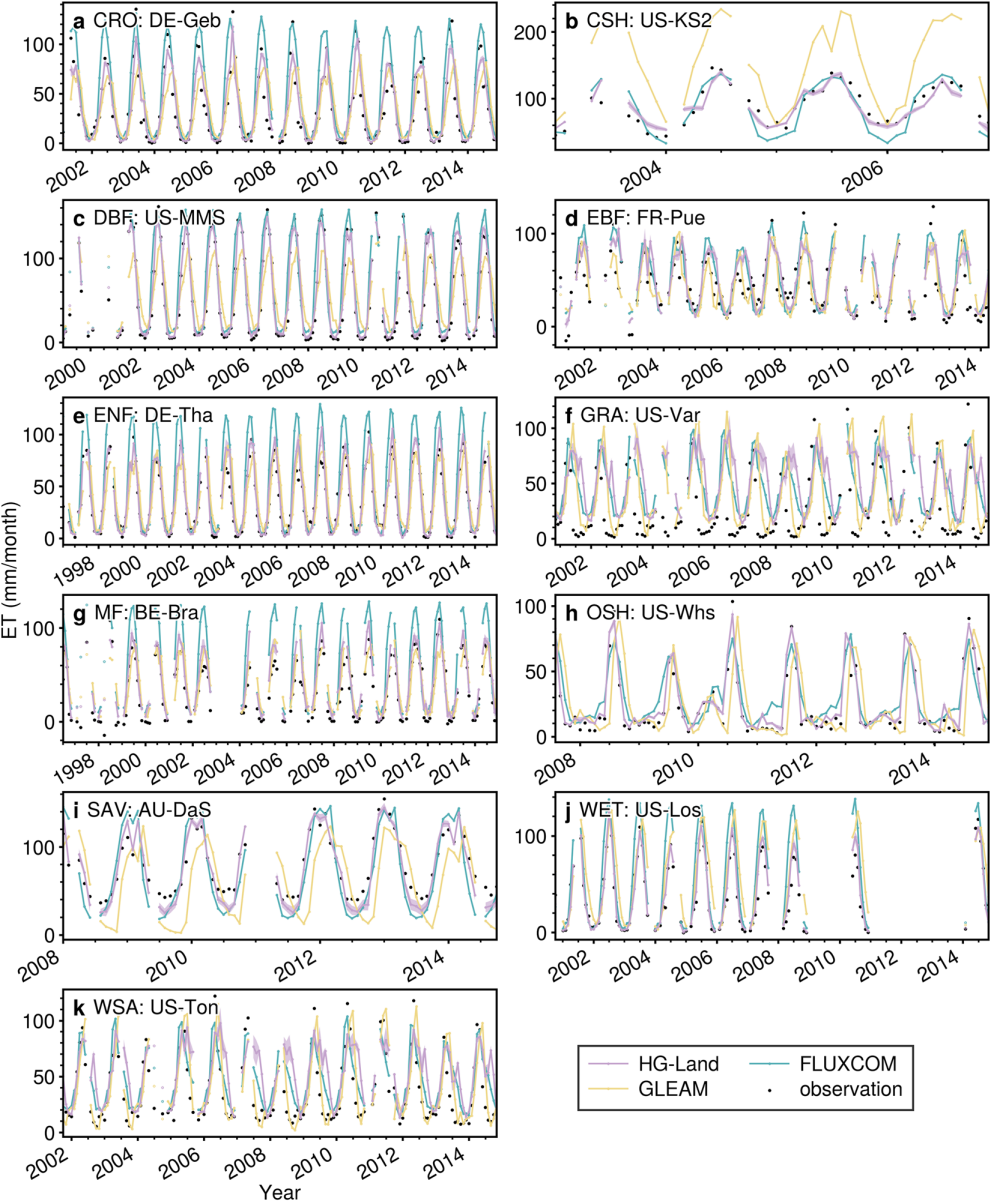
Comparison of ET time series of HG-Land, FLUXCOM, GLEAM, and FLUXNET2015 at 11 flux sites.

Figure 8 presents the ET series for 11 sites with different land types. The shaded region along the HG-Land line represents the estimated uncertainty, indicated by one standard deviation. The HG-Land ET values align closely with the observations, with the ET uncertainty mainly occurring at peak and valley values, evidence of which can be seen in the sites US-Var, AU-DaS, and US-Ton. In contrast, at most other sites, the uncertainty remains minimal. Moreover, the FLUXCOM and GLEAM tend to exhibit overestimations at certain sites. For instance, GLEAM overestimates ET values at the US-KS2 site (Fig. 8b), and FLUXCOM has higher peak values compared to observations at the DE-Tha and BE-Bra sites. Furthermore, all three products fail to capture the lowest observations at the US-Var and BE-Bra sites (Fig. 8f-g).

**Fig. 9:**
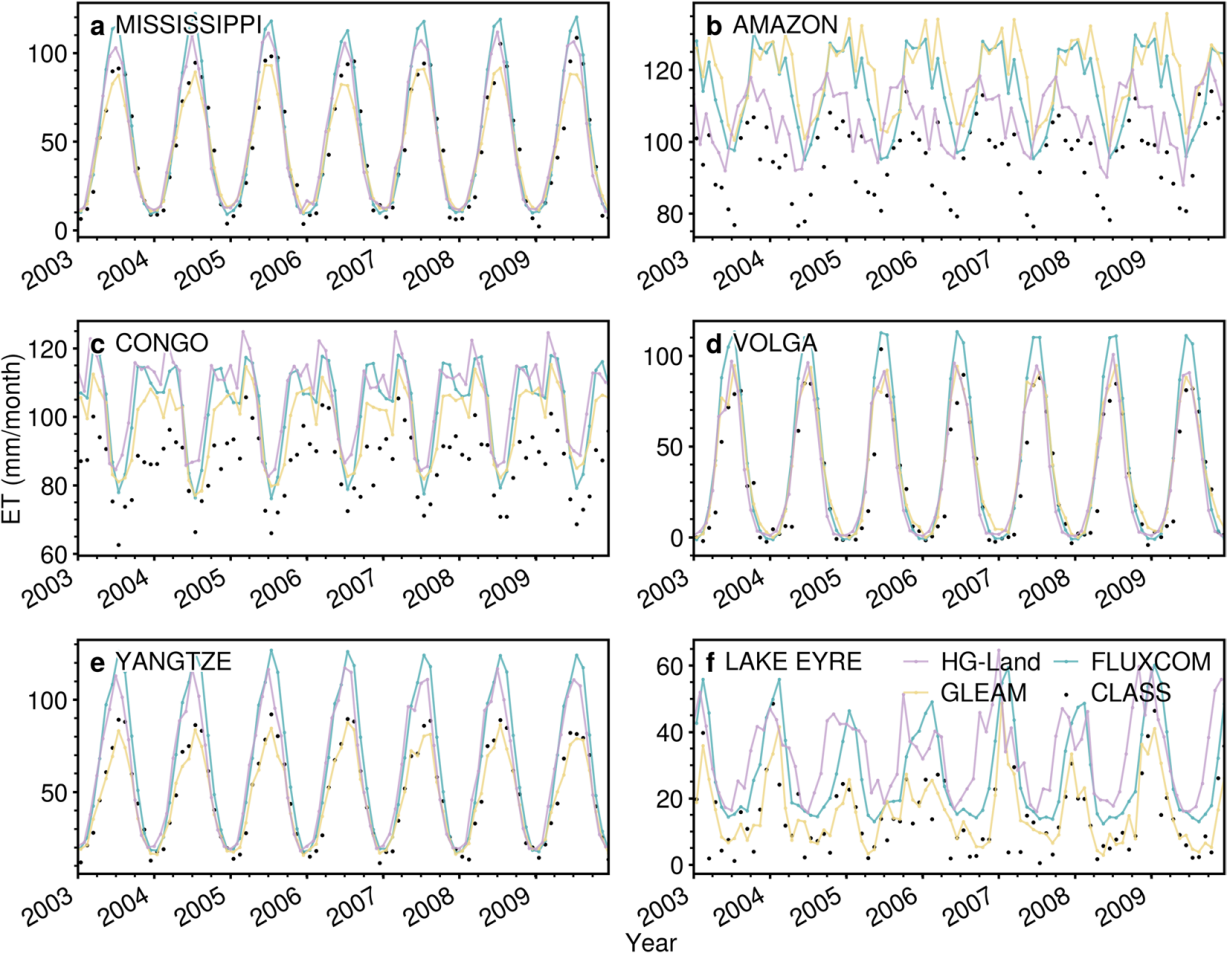
Comparison of ET time series of HG-Land, FLUXCOM, GLEAM, and CLASS over 6 basins.

We then conducted a broader comparison of variations in 6 basins across different continents, as illustrated in Fig. 9. All three products provide accurate estimates for the Mississippi, Volga, and Yangtze basins (Fig. 9a,d,e). However, it becomes apparent that all three products lean towards overestimating ET within both the Amazon and Congo basins (Fig. 9b,c). This highlights an area for potential improvement in the estimation accuracy of these products, particularly within tropical regions.

**Fig. 10:**
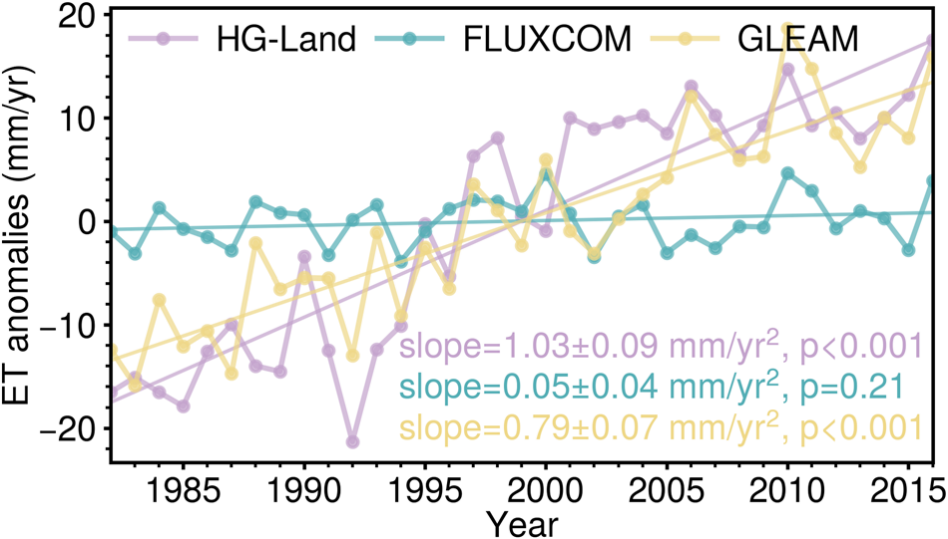
Global annual ET anomalies of HG-Land, FLUXCOM, and GLEAM during 1982–2016.

The global annual ET anomalies during 1982–2016 are shown in Fig. 10, with the ET trends derived through the application of the least square method. The HG-Land exhibits a substantial trend with a value of 1.03 mm/yr^2^, which falls within the range of global ET trend values seen in other products^54^. In contrast, FLUXCOM shows an insignificant trend with a mere value of 0.05 mm/yr^2^. Furthermore, HG-Land and GLEAM demonstrate greater variability when compared to FLUXCOM. Additionally, discernible discrepancies emerge in the inter-annual variabilities among the three products. These disparities can be attributed to variations in the reconstruction methods, where each method selects distinct climate variables or data sources^10^. Consequently, the models may have varying sensitivities to the inter-annual changes of each variable, leading to divergent results.

## Usage Notes

We are pleased to offer a global monthly dataset on ET covering the period 1982–2018. Our dataset demonstrates competitive performance across various scales compared to other existing datasets. However, it is important to acknowledge that our dataset may be subject to relatively high uncertainties in conditions where the land surface classification is not adequately represented in the training set. This is a common challenge encountered by other methods and products due to the homogeneity of ground ET measurements and the inherent heterogeneity of land characteristics. To mitigate this issue, we recommend integrating multiple mainstream datasets to minimize uncertainties and capture more dependable information. Nevertheless, our improved monthly dataset provides a credible reference among current ET products and contributes to expanding our knowledge of ET.

### Supplementary information

Supplementary Information

## Data Availability

The Python code for dataset generation, validation, and visualization is available at https://github.com/FQMei/HG-Land-ET.git.
