# Supplementary material for: Long-term gridded land evapotranspiration reconstruction using Deep Forest with high generalizability
Source: Sci Data. 2023 Dec 18;10:908. doi: 10.1038/s41597-023-02822-8 (PMC10728196; doi:10.1038/s41597-023-02822-8)
Supplement: Supplementary file 1 — Supplementary Information [file 41597_2023_2822_MOESM1_ESM.pdf]

Supplementary Information for

Long-term gridded land evapotranspiration reconstruction using Deep Forest with high generalizability

Qiaomei Feng[a], Junyong Shen[b], Feng Yang[a], Shijing Liang[a], Jiang Liu[b], Xingxing Kuang[a], Dashan Wang[a,*], Zhenzhong Zeng[a,*]

[a] School of Environmental Science and Engineering, Southern University of Science and Technology, Shenzhen 518055, China

[b] Department of Computer Science and Engineering, Southern University of Science and Technology, Shenzhen 518055, China

[*] Correspondence to: zengzz@sustech.edu.cn (Zhenzhong Zeng)

Mailing Address: College of Engineering N808, Southern University of Science and Technology, Shenzhen, China

Content

Supplementary methods 1

Supplementary results 3

Supplementary Tables S1-S4 4

Supplementary Figures S1-S14 8

Uncertainties associated with the sources of forcing datasets 8

Model interpretability 14

Comparison with existing products 17

Supplementary methods

Many studies have suggested that the inconsistent ET estimates across various ET datasets can in part, be ascribed to the selection of forcing datasets. For example, Badgley et al. underscored the prominence of the net radiation dataset as a key driver of such discrepancies[1]. While others have indicated that uncertainties in ET estimations might arise from the sources of meteorological and LAI data[2]. To assess the influence of variations in forcing data sources on the predictions of the Deep Forest model, here we conducted an investigation by substituting the original input data with information from either gridded datasets or *in-situ* observations.

In the context of gridded datasets, we replaced the sources of eight meteorological and vegetation variables with data derived from NCEP/DOE Reanalysis 2 provided by NOAA PSL[3] and AVHRR GIMMS LAI3g and FAPAR3g dataset[4] (Table S1). The rationale behind this selection is rooted in the fact that NCEP/DOE Reanalysis 2 offers a comprehensive array of input features, thereby ensuring a higher degree of consistency of input datasets. In addition, we have incorporated AVHRR GIMMS LAI and FAPAR v5.0, which provide a long-term dataset covering the entire study period and exhibit a fine resolution of approximately 0.083°.

While incorporating inputs derived from *in-situ* observations, we leveraged the FLUXNET2015 dataset to extract essential forcing variables, encompassing elements such as air temperature, precipitation, net radiation, wind speed, and atmospheric pressure (Table S2). The vapor pressure and potential ET input variables were calculated using meteorological variables from the FLUXNET2015 dataset. Specifically, the vapor pressure was computed using vapor pressure deficit and air temperature data from the FLUXNET2015 dataset, as calculated by Eqs. (1):

$$VAP = 6.108exp(\frac{17.27T}{T + 237.3}) - VPD \tag{1}$$

where VAP is vapor pressure (hPa); T is air temperature (°C); and VPD represents vapor pressure deficit (hPa). Additionally, the potential ET was determined using the updated Penman-Monteith equation (Eqs. (2)) provided by FAO (Food and Agricultural Organization)[5]:

$$PET = \frac{0.408\Delta(R_n - G) + \gamma \frac{900}{T + 273.16} U_2(e_a - e_d)}{\Delta + \gamma(1 + 0.34U_2)} \tag{2}$$

$$\Delta = \frac{4098[0.6108exp(\frac{17.27T}{T + 237.3})]}{(T + 237.3)^2} \tag{3}$$

$$\gamma = 0.665 \times 10^{-3}P \tag{4}$$

$$U_2 = U_{10}\frac{\ln(128)}{\ln(661.3)} \tag{5}$$

where PET is grass reference ET (mm/d); Δ is the slope of the vapor pressure curve (kPa/°C), calculated using Eqs. (3); R_n is the net radiation (MJ/ (m^2 d)); G is the soil heat flux (MJ/ (m^2 d)); γ is the psychrometric constant (kPa/°C), estimated using Eqs. (4); P is the atmospheric pressure (kPa); T is the 2 m mean temperature (°C); U_2 is the 2 m wind speed (m/s), converted from 10 m wind speed using Eqs. (5); (e_a-e_d) is the vapor pressure deficit measured at 2 m height (kPa).

For model training, the testing set comprises 20% of the total samples, including all samples from sites located in regions with a CRO (croplands) land type, along with randomly selected samples that do not belong to the CRO land cover. This partitioning strategy was employed to assess the model's performance both spatially and temporally, providing a fair evaluation across various forcing datasets. The remaining 80% of the data was used for training.

Supplementary results

We introduced alternative gridded datasets as input sources (Table S1) and subsequently assessed the disparities in ET estimations. Remarkably, the model's performance in the testing set exhibited a slight decline (Fig. S1). The statistical metrics RMSE and MAE increased from their initial values of 21.55 mm/month and 14.51 mm/month to 23.29 mm/month and 15.71 mm/month, respectively. These changes suggest that the Deep Forest algorithm demonstrates a restricted sensitivity to variations in gridded input datasets, and the resulting discrepancies remain within acceptable ranges.

Furthermore, when employing *in-situ* data as inputs, the retrained model exhibited marginal improvements in accuracy (Fig. S2). This led to the attainment of R, RMSE, and MAE values of 0.90, 22.66 mm/month, and 14.42 mm/month, respectively. Given the acknowledged precision associated with *in-situ* measurements, this result underscores the capability of the selected forcing datasets to faithfully mirror the land surface conditions. Therefore, we reported the model outputs derived from the input data described in Table 1, while using the results from the alternative gridded datasets (Table S1) and *in-situ* observations (Table S2) to assess the uncertainty associated with the forcing dataset.

Supplementary Tables S1-S4

Table S1. Alternative gridded forcing datasets for model training.

<table>
<tr><th>Variables</th><th>Original data source</th><th>Alternative data source</th><th>Function</th></tr>
<tr><td>fAPAR (-)
LAI (m^2/ m^2)</td><td>NOAA CDR AVHRR LAI and FAPAR v5.0</td><td>AVHRR GIMMS LAI3g and FAPAR3g</td><td>biological information</td></tr>
<tr><td>2 m temperature (tmp, degree Celsius)
precipitation (pre, mm/month)</td><td>CRU TS v4.05</td><td rowspan="2">NCEP/DOE Reanalysis 2</td><td rowspan="3">meteorological information</td></tr>
<tr><td>surface net solar radiation (rad, J/m^2)
10 m wind speed (wnd, m/s)
skin temperature (tsk, K)
surface pressure (prs, Pa)</td><td>ERA5-Land</td></tr>
<tr><td>vapour pressure (vap, hPa)
potential ET (pet, mm/day)
wet days (wet, days/month)
frost days (frs, days/month)</td><td colspan="2">CRU TS v4.05</td></tr>
</table>

Table S2. Alternative *in-situ* forcing datasets for model training.

Variables	Original data source	*In-situ* observed data source	Function
potential ET (pet, mm/day)<br>vapour pressure (vap, hPa)<br>2 m temperature (tmp, degree Celsius)<br>precipitation (pre, mm/month)	CRU TS v4.05	FLUXNET2015	meteorological information
surface net solar radiation (rad, J/m2)<br>10 m wind speed (wnd, m/s)<br>surface pressure (prs, Pa)	ERA5-Land		
wet days (wet, days/month)<br>frost days (frs, days/month)	CRU TS v4.05		
skin temperature (tsk, K)	ERA5-Land		
fAPAR (-)<br>LAI (m^2/ m^2)	NOAA CDR AVHRR LAI and FAPAR v5.0		biological information

Table S3. Climate classification based on Aridity Index provided by UNEP[6].

Aridity index	Climate type
AI<0.05	Hyper-arid
0.05≤AI<0.2	Arid
0.2≤AI<0.5	Semi-arid
0.5≤AI<0.65	Dry Subhumid
AI≥0.65	Humid

Table S4. Site information for SHAP calculation.

Site ID	Latitude (°)	Longitude (°)	IGBP type	Aridity index	Climate type
BE-Vie	50.3049	5.9981	MF	1.3845	Humid
BR-Sa3	-3.018	-54.9714	EBF	1.4204	Humid
CA-Qfo	49.6925	-74.3421	ENF	1.3811	Humid
CA-TP3	42.7068	-80.3483	ENF	0.9912	Humid
CG-Tch	-4.2892	11.6564	SAV	1.0802	Humid
CH-Fru	47.1158	8.5378	GRA	1.7602	Humid
CN-Cha	42.4025	128.0958	MF	0.7656	Humid
IT-Isp	45.8126	8.6336	DBF	1.1475	Humid
IT-PT1	45.2009	9.061	DBF	0.7581	Humid
PA-SPs	9.3138	-79.6314	GRA	1.5052	Humid
AU-Cum	-33.6152	150.7236	EBF	0.5223	Dry Subhumid
AU-DaS	-14.1593	131.3881	SAV	0.4811	Semi-arid
AU-Gin	-31.3764	115.7138	WSA	0.3205	Semi-arid
AU-Stp	-17.1507	133.3502	GRA	0.2322	Semi-arid
AU-TTE	-22.287	133.64	GRA	0.0850	Arid
CA-SF1	54.485	-105.818	ENF	0.6154	Dry Subhumid
ES-Amo	36.8336	-2.2523	OSH	0.1291	Arid
US-Atq	70.4696	-157.409	WET	0.4344	Semi-arid
US-SRC	31.9083	-110.84	OSH	0.1568	Arid
US-Twt	38.1087	-121.653	CRO	0.2178	Semi-arid

Supplementary Figures S1-S14

Uncertainties associated with the sources of forcing datasets

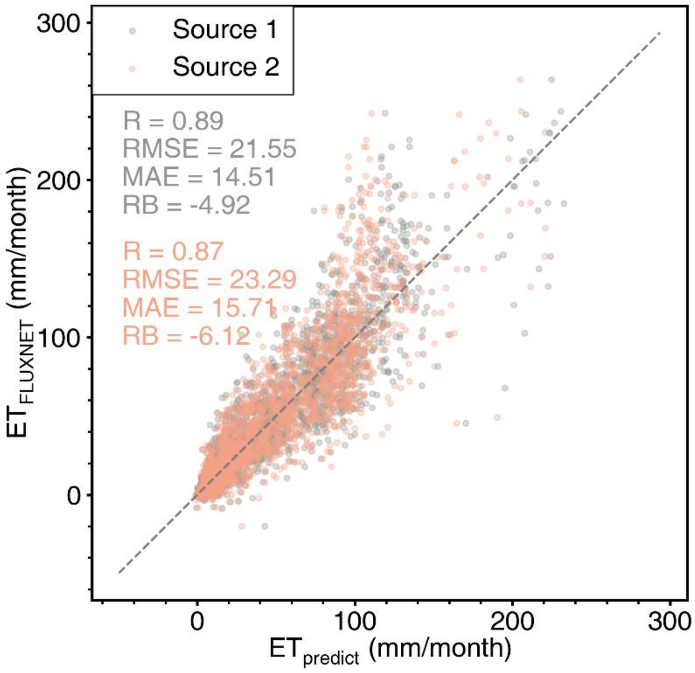

Fig. S1. Model accuracy comparison using two sets of gridded forcing data sources. Source 1 (Original data source): CRU TS v4.05, ERA5-Land, and NOAA CDR AVHRR LAI and FAPAR v5.0; Source 2 (Alternative data source): CRU TS v4.05, NCEP/DOE Reanalysis 2, and AVHRR GIMMS LAI3g and FAPAR3g. Refer to Table S1 for dataset information. The testing set contains 2,195 samples.

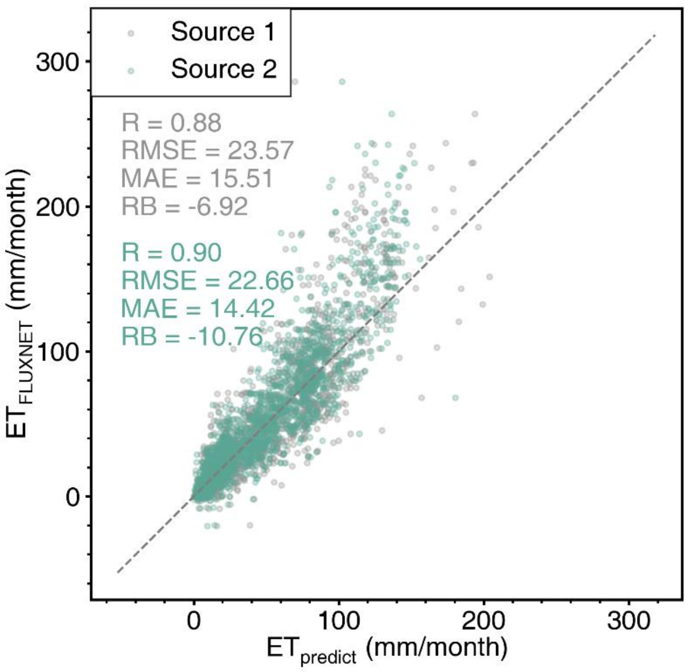

Fig. S2. Model accuracy comparison using gridded and site-level forcing data sources, respectively. Source 1 (Original data source): CRU TS v4.05, ERA5-Land, and NOAA CDR AVHRR LAI and FAPAR v5.0; Source 2 (*In-situ* observed data source): FLUXNET2015 meteorological observations, CRU TS v4.05, ERA5-Land, NOAA CDR AVHRR LAI and FAPAR v5.0. Refer to Table S2 for dataset information. The testing set contains 1,722 samples, following the removal of missing values in the *in-situ* forcing dataset.

Model development

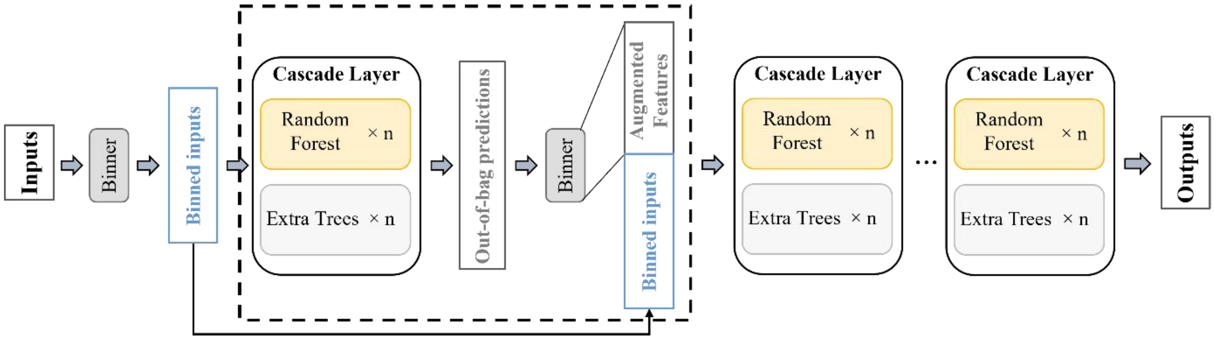

Fig. S3. The architecture of the Deep Forest model, adapted from Zhou and Feng[7]. *Binner* is a common preprocessing step used to discretize continuous features and reduce the impact of outliers. The binned training data, i.e., *Binned inputs*, is fed into the first *Cascade Layer* which consists of multiple random forests and an equal number of extra trees. The predictions of the out-of-bag samples from the first *Cascade Layer* served as *augmented features* to concatenate with the *Binned inputs* and passed as inputs to the next *Cascade Layer*. The process in the dotted box is repeated iteratively, with additional Cascade Layers created if the performance of the current layer is better than the previous layers.

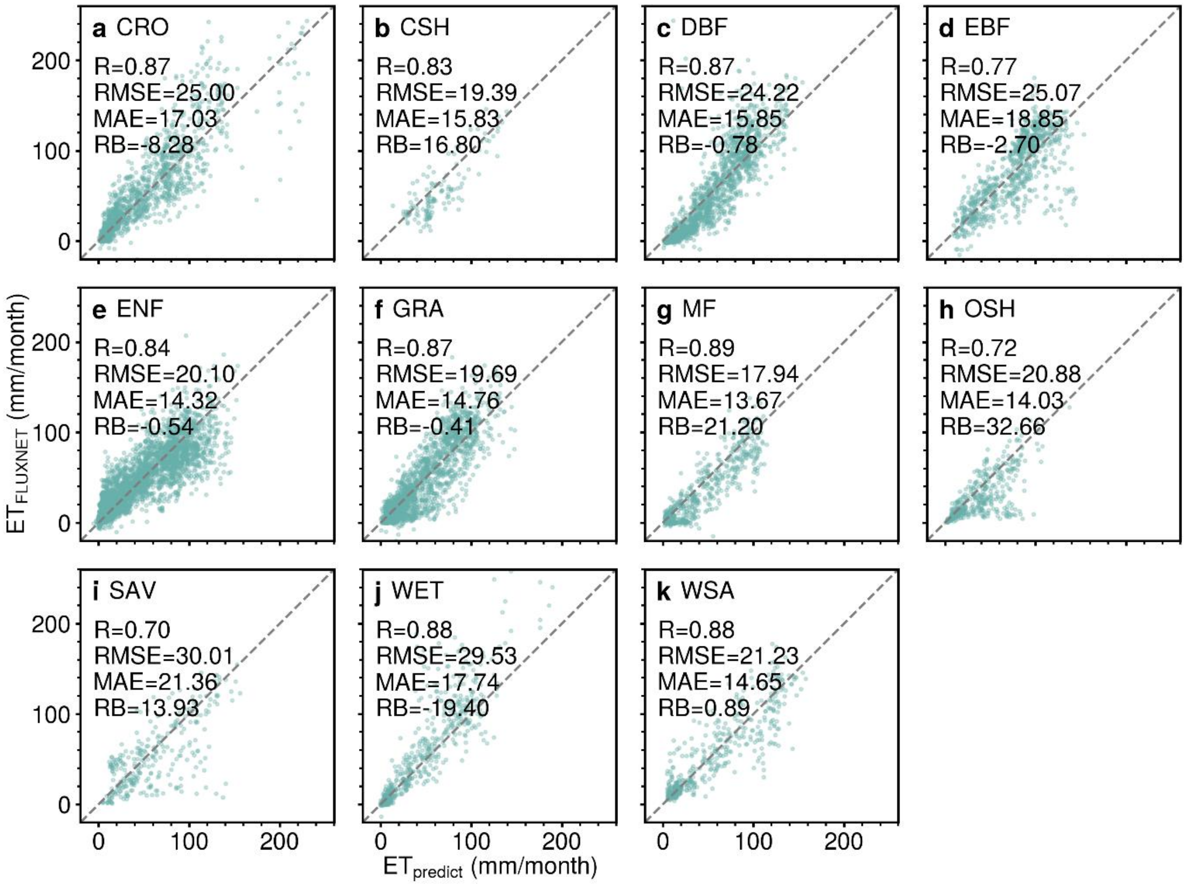

Fig. S4. The accuracy of 11 distinct models on their respective testing sets. Each testing set comprises a single surface land classification labeled in the upper left of the panels.

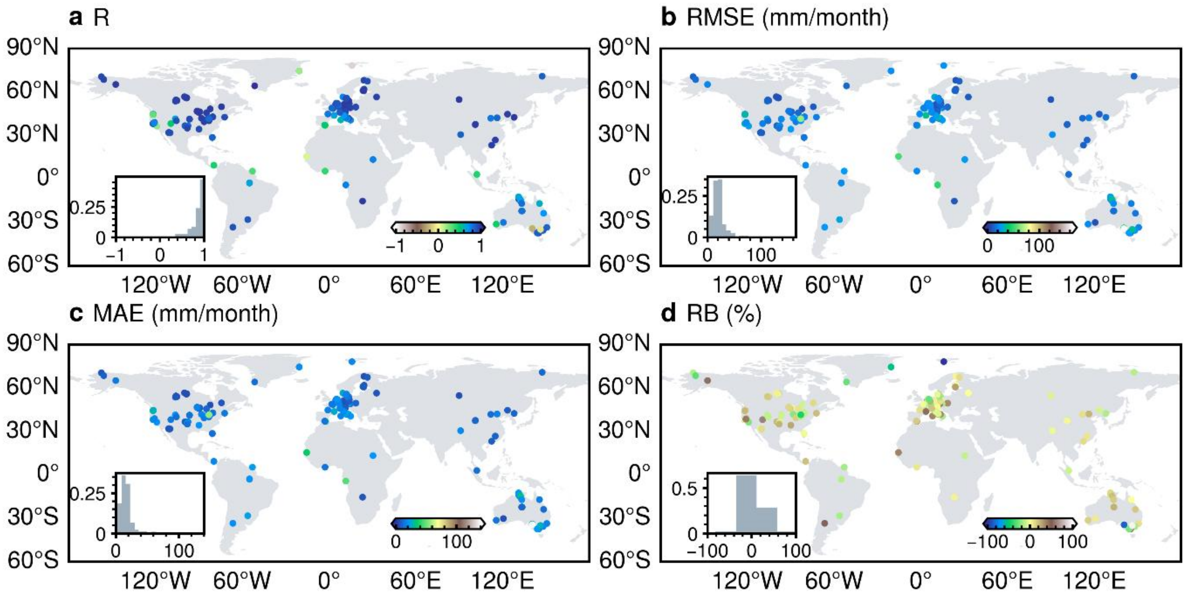

Fig. S5. Spatial variability of ET accuracy across different sites, derived from 11 distinct model outputs compared to FLUXNET observations. (a) R; (b) RMSE; (c) MAE; (d) RB. The inset figure shows the frequency distribution histogram of each metric. The flux sites US-Wi1, US-Wi9, and US-Wi7 were omitted from the figure due to a limited availability of valid data records from these sites.

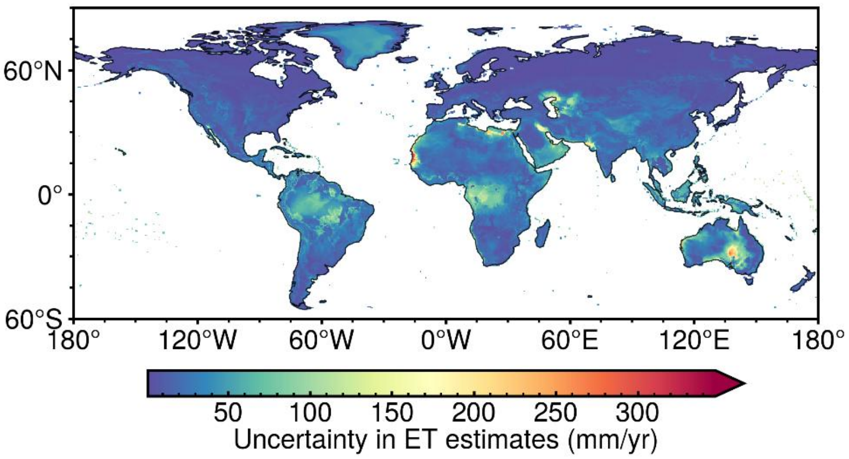

Fig. S6. Global distribution of uncertainties in ET estimates resulting from model extrapolation. The monthly ET estimates generated by models were first aggregated into multi-year mean values for the period 1982-2018, respectively. The uncertainties were calculated among these multi-year values.

Model interpretability

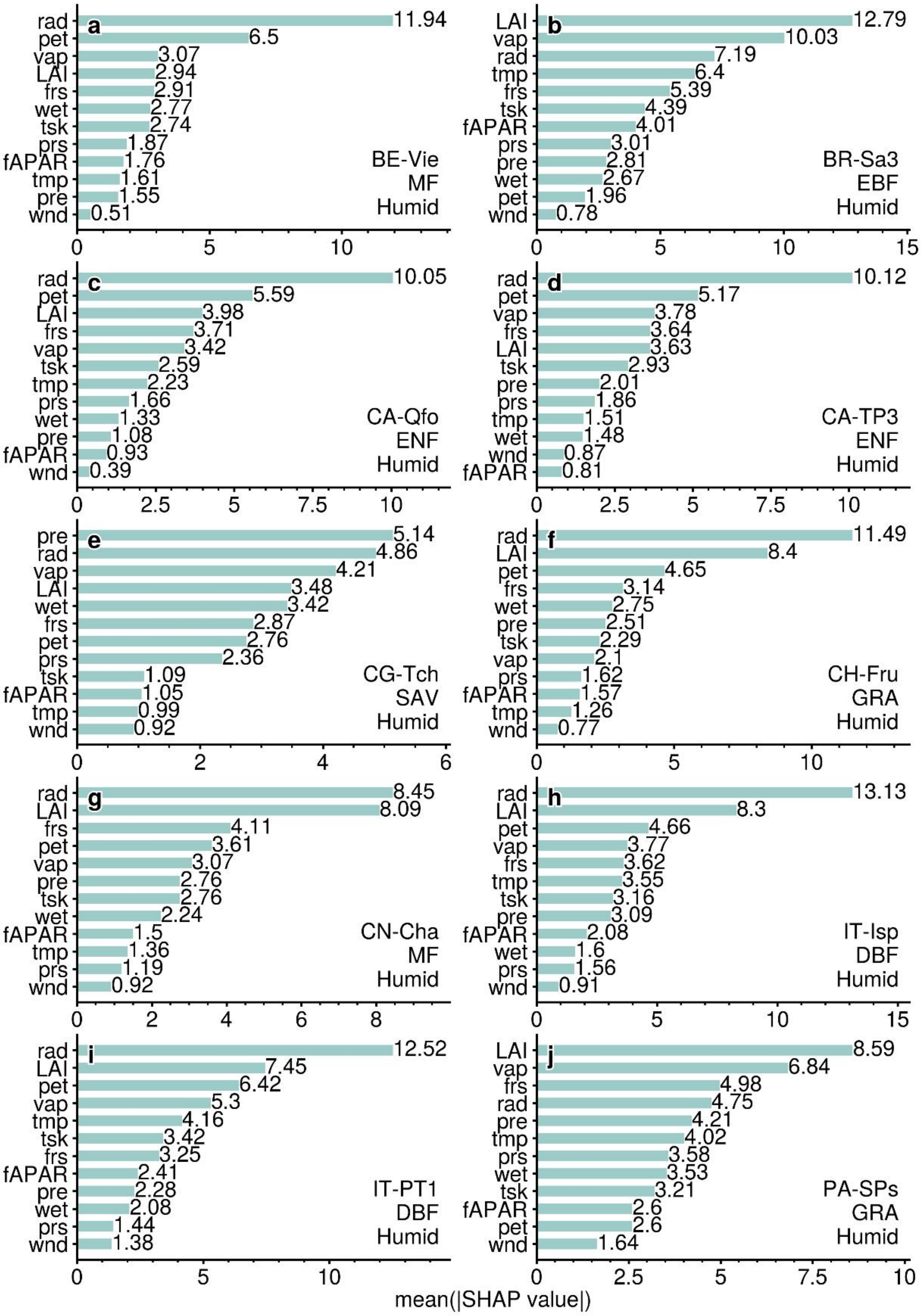

Fig. S7. Mean absolute SHAP values for features at selected sites located in humid regions. The inset text in the lower right of each panel shows the site ID, its land cover classification, and the climate classification.

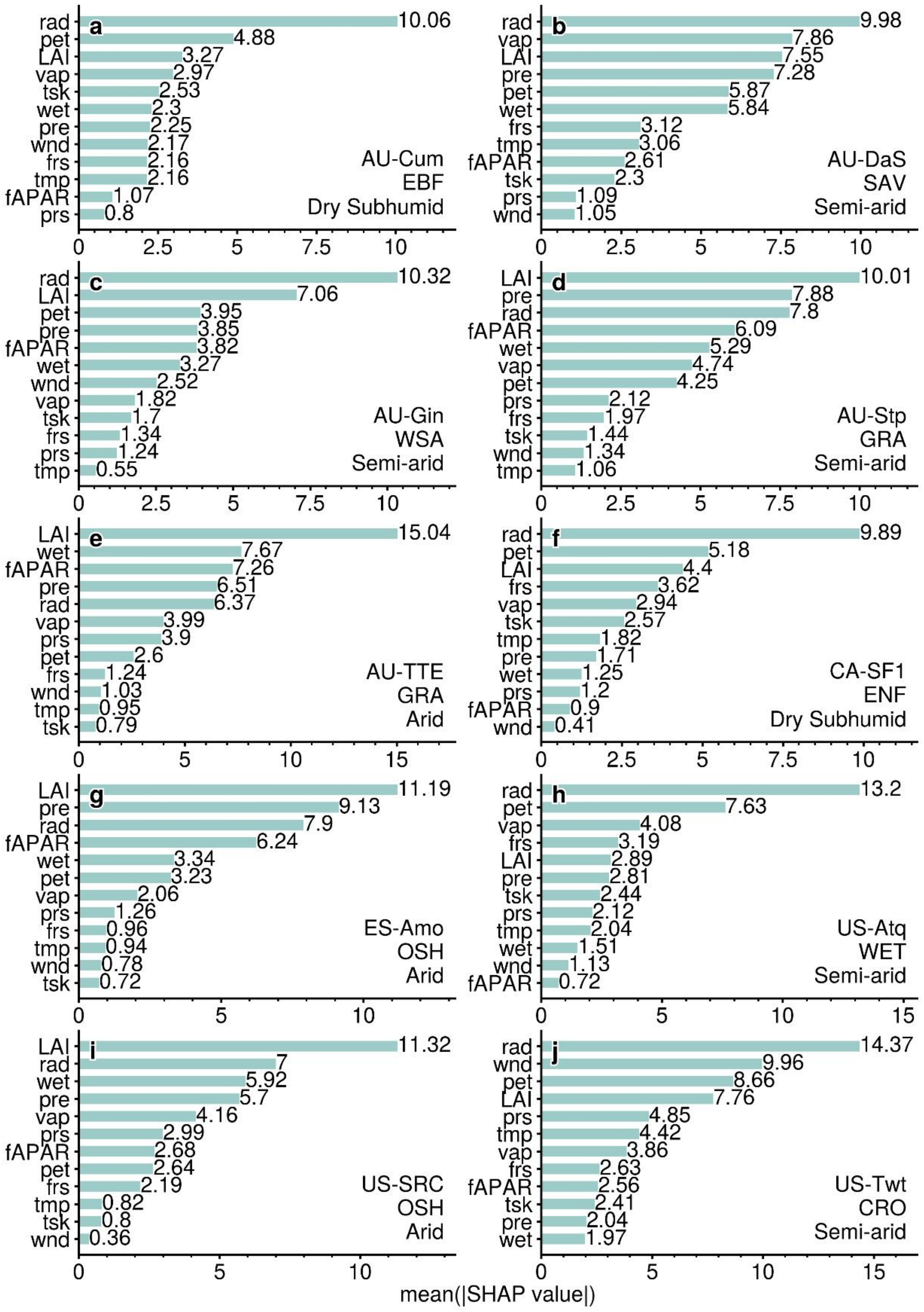

Fig. S8. Mean absolute SHAP values for features at selected sites located in dry regions. The inset text in the lower right of each panel shows the site ID, its land cover classification, and the climate classification.

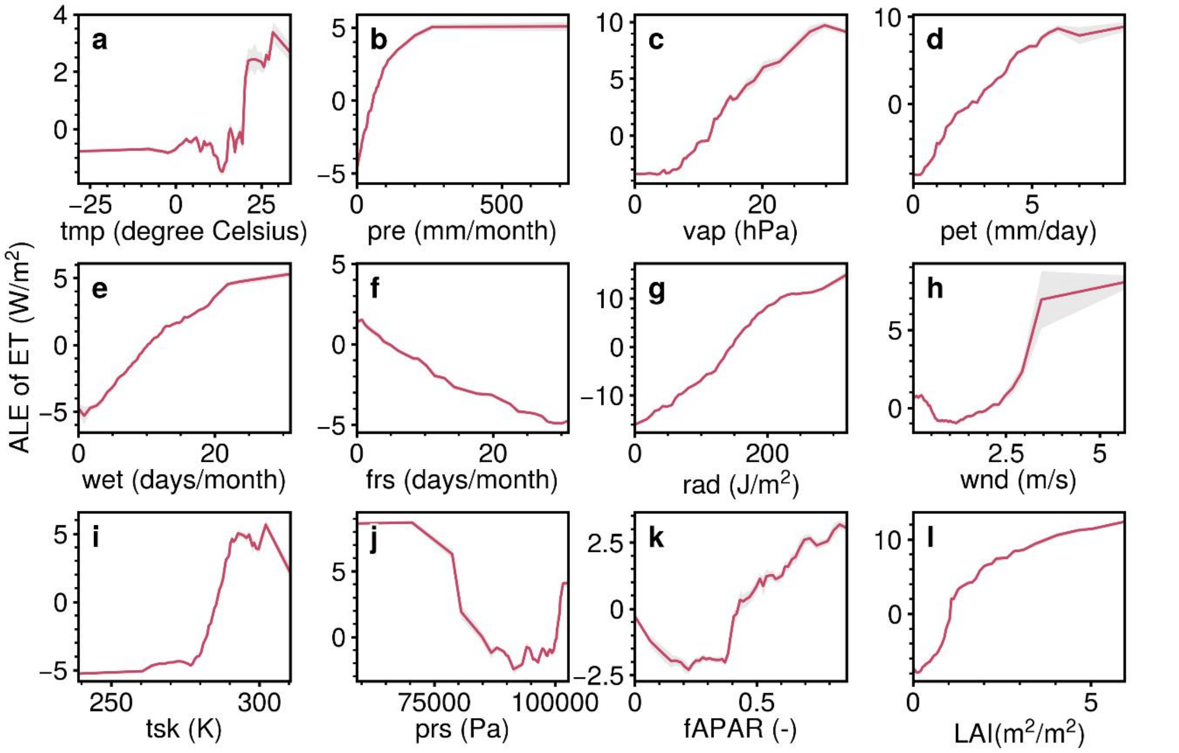

Fig. S9. Impacts of variables on ET estimations. (a) tmp (degree Celsius), (b) pre (mm/month), (c) vap (hPa), (d) pet (mm/day), (e) wet (days/month), (f) frs (days/month), (g) rad (J/m^2), (h) wnd (m/s), (i) tsk (K), (j) prs (Pa), (k) fAPAR (-), and (l) LAI ($m^{2/}m^2$). The gray shading represents the 95% confidence interval of the effects. ET values were centered to zero based on the ALE theory[8].

Comparison with existing products

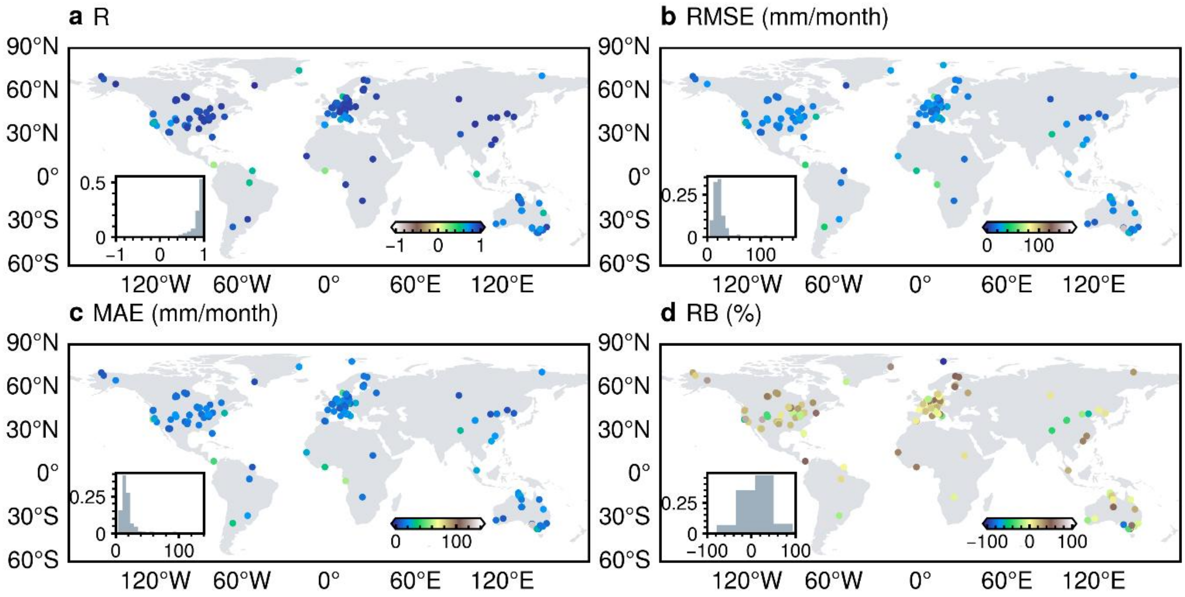

Fig. S10. Spatial variability of FLUXCOM ET accuracy across different sites compared to FLUXNET observations. (a) R; (b) RMSE; (c) MAE; (d) RB. The inset figure shows the frequency distribution histogram of each metric. The flux sites US-Wi1, US-Wi9, and US-Wi7 were omitted from the figure due to a limited availability of valid data records from these sites.

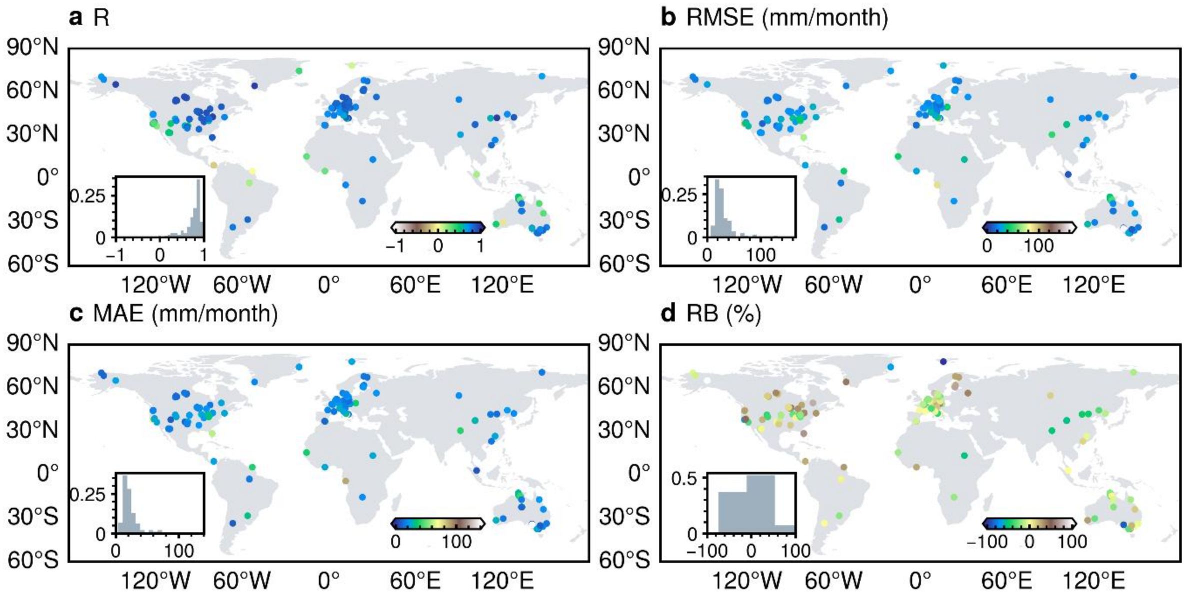

Fig. S11. Spatial variability of GLEAM ET accuracy across different sites compared to FLUXNET observations. (a) R; (b) RMSE; (c) MAE; (d) RB. The inset figure shows the frequency distribution histogram of each metric. The flux sites US-Wi1, US-Wi7, US-Wi9, IT-Bci, and IT-Noe were omitted from the figure due to a limited availability of valid data records from these sites or missing values in the GLEAM dataset.

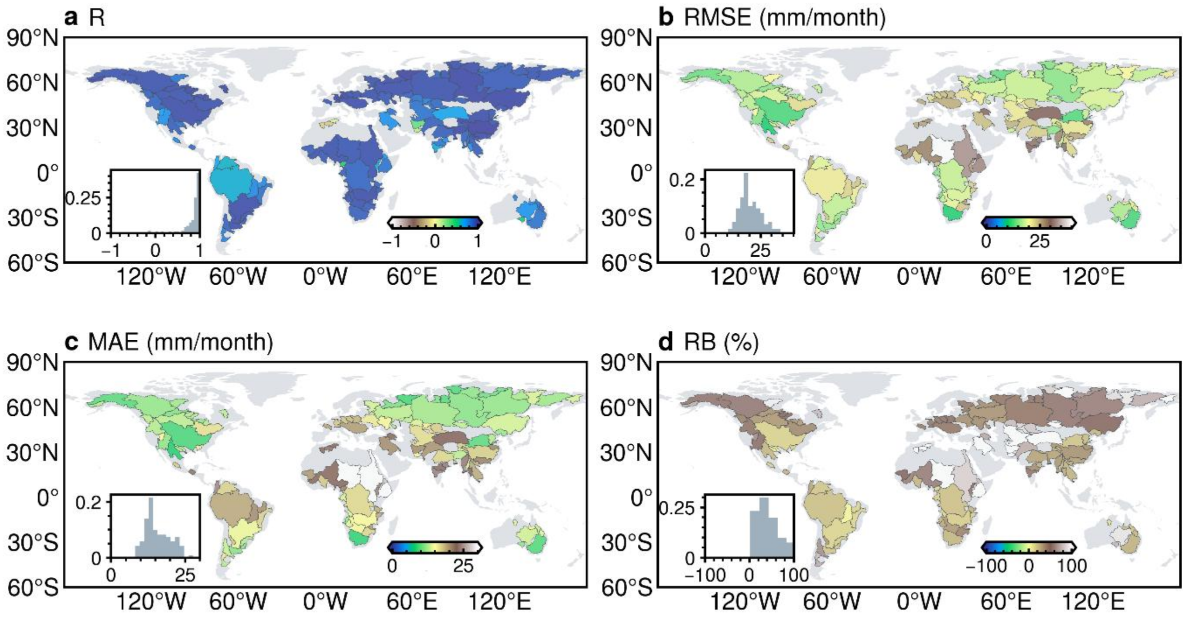

Fig. S12. Spatial variability of FLUXCOM ET accuracy over basins compared to CLASS ET: (a) R; (b) RMSE; (c) MAE; (d) RB. The inset figure shows the frequency distribution histogram of each metric.

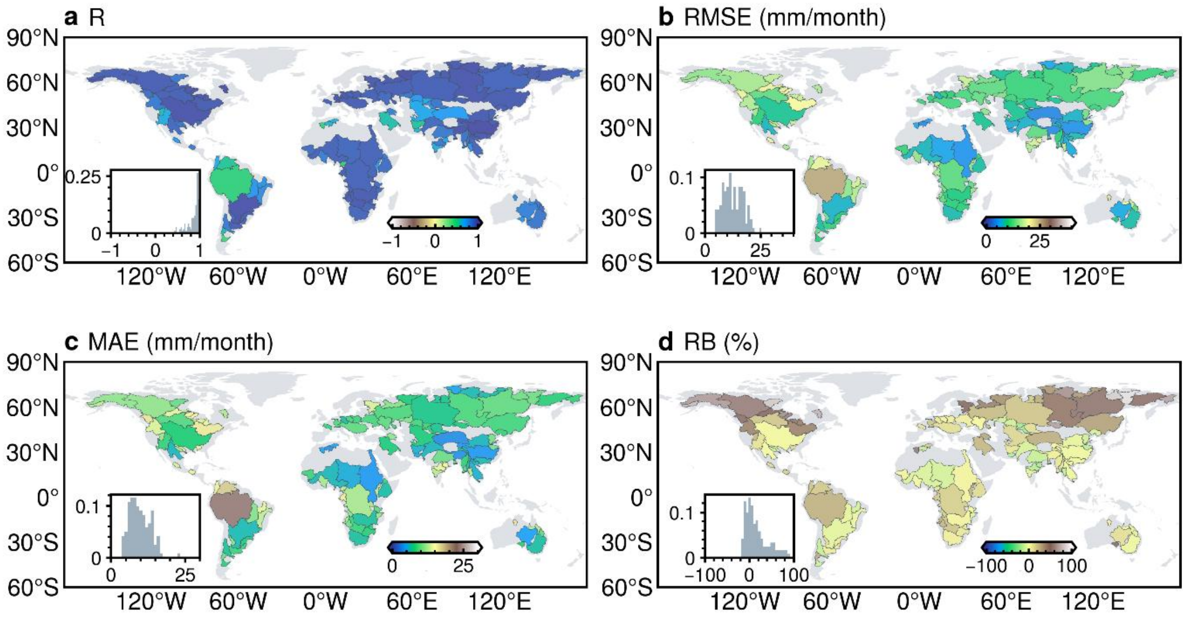

Fig. S13. Spatial variability of GLEAM ET accuracy over basins compared to CLASS ET. (a) R; (b) RMSE; (c) MAE; (d) RB. The inset figure shows the frequency distribution histogram of each metric.

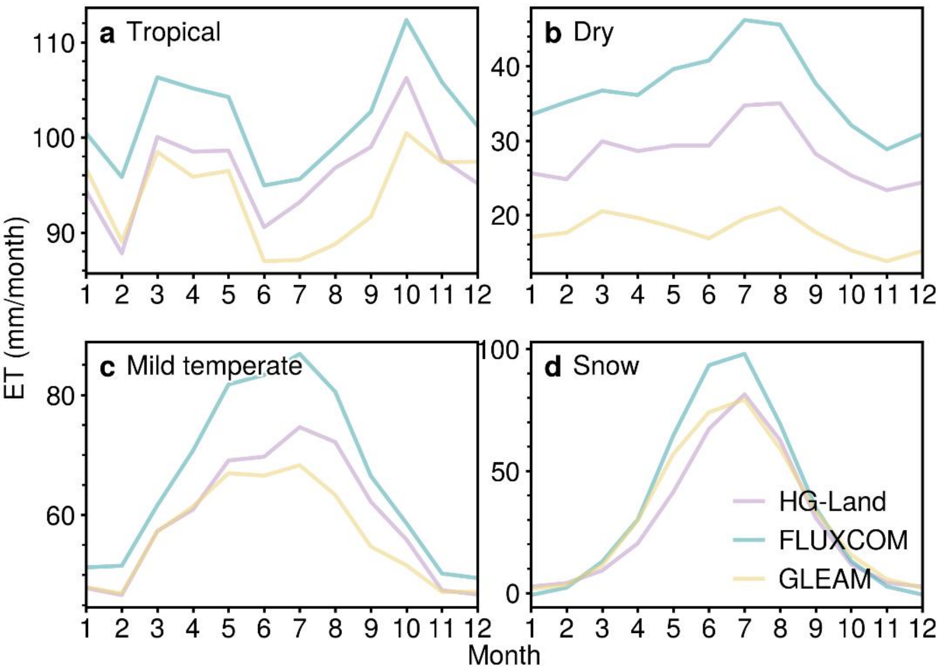

Fig. S14. Seasonal variations of HG-Land, FLUXCOM, and GLEAM among four major Köppen climate classifications: (a) tropical, (b) dry, (c) mild temperate, and (d) snow climates.

Reference:

1. Badgley, G., Fisher, J. B., Jiménez, C., Tu, K. P. & Vinukollu, R. On uncertainty in global terrestrial evapotranspiration estimates from choice of input forcing datasets. *J. Hydrometeorol.* **16**, 1449–1455 (2015).
2. Chen, H. *et al.* Evaluation of evapotranspiration models using different LAI and meteorological forcing data from 1982 to 2017. *Remote Sens.* **12**, 2473 (2020).
3. Kanamitsu, M. *et al.* NCEP–DOE AMIP-II Reanalysis (R-2). *Bull. Am. Meteorol. Soc.* **83**, 1631–1644 (2002).
4. Zhu, Z. *et al.* Global data sets of vegetation Leaf Area Index (LAI)3g and Fraction of Photosynthetically Active Radiation (FPAR)3g derived from Global Inventory Modeling and Mapping Studies (GIMMS) Normalized Difference Vegetation Index (NDVI3g) for the period 1981 to 2011. *Remote Sens.* **5**, 927–948 (2013).
5. Allen, R. G., Smith, M., Pereira, L. S. & Perrier, A. An update for the calculation of reference evapotranspiration. *ICID bulletin* **43**, 35 (1994).
6. United Nations Environment Program. *World Atlas of Desertification: Second Edition*. (1997).
7. Zhou, Z.-H. & Feng, J. Deep forest. *Natl. Sci. Rev.* **6**, 74–86 (2019).
8. Apley, D. W. & Zhu, J. Visualizing the effects of predictor variables in black box supervised learning models. *J. R. Stat. Soc. Series B Stat. Methodol.* **82**, 1059–1086 (2020).
